# Teneurins assemble into presynaptic nanoclusters that promote synapse formation via postsynaptic non-teneurin ligands

**DOI:** 10.1038/s41467-022-29751-1

**Published:** 2022-04-28

**Authors:** Xuchen Zhang, Pei-Yi Lin, Kif Liakath-Ali, Thomas C. Südhof

**Affiliations:** 1 Howard Hughes Medical Institute, Stanford University, Stanford, CA USA; 2 Department of Molecular and Cellular Physiology, Stanford University, Stanford, CA USA

**Keywords:** Molecular neuroscience, Neural circuits

## Abstract

Extensive studies concluded that homophilic interactions between pre- and postsynaptic teneurins, evolutionarily conserved cell-adhesion molecules, encode the specificity of synaptic connections. However, no direct evidence is available to demonstrate that teneurins are actually required on both pre- and postsynaptic neurons for establishing synaptic connections, nor is it known whether teneurins are localized to synapses. Using super-resolution microscopy, we demonstrate that Teneurin-3 assembles into presynaptic nanoclusters of approximately 80 nm in most excitatory synapses of the hippocampus. Presynaptic deletions of Teneurin-3 and Teneurin-4 in the medial entorhinal cortex revealed that they are required for assembly of entorhinal cortex-CA1, entorhinal cortex-subiculum, and entorhinal cortex-dentate gyrus synapses. Postsynaptic deletions of teneurins in the CA1 region, however, had no effect on synaptic connections from any presynaptic input. Our data suggest that different from the current prevailing view, teneurins promote the establishment of synaptic connections exclusively as presynaptic cell-adhesion molecules, most likely via their nanomolar-affinity binding to postsynaptic latrophilins.

## Abstract (web summary)

Teneurins are cell-adhesion molecules that help form synaptic connections between neurons. Here the authors demonstrate that teneurins on the presynaptic side are essential to form connections.

## Introduction

Teneurins are evolutionarily conserved adhesion molecules that are expressed in multiple tissues, but are present at the highest levels in brain^1,2^. Vertebrate genomes include four teneurin genes (gene symbols in mice: *Tenm1* to *Tenm4*), whereas arthropods contain only two teneurin genes, and other invertebrates only a single teneurin gene^3,4^. Teneurins are type II transmembrane proteins containing an N-terminal cytoplasmic sequence (~320 residues), a single transmembrane region, and a very large extracellular sequence (~2390 residues)^3,5^. As revealed by the atomic structures of human^6,7^ and chicken Teneurin-2 (*Tenm2*)^8,9^, the extracellular teneurin sequences are composed of an unusual set of domains, comprising 8 juxtamembranous EGF-like repeats, an Ig-like domain, a β-propeller domain, a β-barrel domain, a toxin-like domain, and a C-terminal sequence resembling corticotropin-releasing factor^6–9^. Teneurins form obligatory homo- and heterodimers in a cis-configuration during their transport to the cell surface because their 2nd and 5th EGF-like repeats have only 5 cysteine residues and form covalent disulfide-bonded dimers^3,5^. Among teneurins domains, the β-barrel domain is the largest, and serves as a scaffold for the toxin-like domain. The Ig-like domain of teneurins forms a ‘plug’ at the N-terminus of the β-barrel domain, while the β-propeller domain sticks out to the side. Latrophilins, which are postsynaptic adhesion GPCRs that represent the only currently known high-affinity ligands for teneurins^10–12^, bind to the β-barrel domain of all teneurins^7,8^.

Teneurins are most highly expressed in neurons, but are also detected in other tissues. Teneurins were linked to several genetic disorders in humans, such as anosmia^13^, essential tremor^14^, microphthalmia^15^, Parkinson’s disease^16^, bipolar disorder^17^, and schizophrenia^18^. During embryonic development, Teneurin-4 (*Tenm4*) is required for gastrulation and mesoderm induction^19,20^. In the brain*Tenm4* is essential for the development of type I/II oligodendrocytes^21,22^ and astrocytes^23^, while *Tenm2* is important for neuronal migration^8^. Teneurin-1 (*Tenm1*), moreover, is highly expressed in muscle where it is required for the normal organization of muscle fibers^24^.

Most efforts on teneurins, however, have focused on the brain, in particular on the role of teneurin-3 (*Tenm3*) in shaping synaptic connections. In mice*Tenm3* is required for the formation of proper synaptic projections from the retina to the lateral geniculate nucleus^25^ and the superior colliculus^26^, from the geniculate nucleus to the visual cortex^27^, from the thalamus to the striatum^28^, and from the proximal CA1 to the distal subiculum^29^. Moreover, Tenm2 is also essential for the formation of synaptic projections from the retina to the lateral geniculate nucleus and superior colliculus^30^. These studies were mostly performed in constitutive KO mice, and did not reveal whether the observed phenotypes represent an impairment in axonal pathfinding or synapse formation, especially since the localization of *Tenm3* protein was not examined.

In order to explain the impairments in synaptic connectivity in *Tenm3* KO mice, a widely accepted hypothesis posits that teneurins function as homophilic adhesion molecules, and that their homophilic interactions confer specificity to synaptic connections^1^. This hypothesis was motivated by the finding that transfection of *Tenm2* in cultured cells makes these cells clump together^31^, and was supported by the observation that *Tenm1* and *Tenm3* are expressed in interconnected regions of the brain^32^, especially the visual system^25^ and the hippocampus^29^. Moreover, overexpression of teneurins in *Drosophila* (which has only two teneurin genes)^33,34^ and axon mapping experiments in mice^29^ supported the hypothesis that homophilic interactions between teneurins confer synaptic specificity onto neuronal circuits.

The notion that homophilic interactions between teneurins control synaptic specificity is a very attractive hypothesis^1^, but several observations give rise to caution. First, cell clumping as a function of teneurin overexpression is observed only in a few types of tissue culture cells^29,31^. For example, in HEK293 cells that are commonly used to monitor the function of cell-adhesion molecules, teneurins do not engage in homophilic interactions, but robustly mediate heterophilic interactions by binding to latrophilins^10,35^. Second, teneurins do not exhibit specificity in homophilic binding assays. The clumping observed with some teneurin-expressing cells appears to work in all combinations of teneurin isoforms^36^. It is difficult to imagine how non-specific homophilic trans-interactions could encode synapse specificity. Third, in mammalian brain only *Tenm1* and *Tenm3* exhibit a strikingly discrete expression pattern in interconnected neurons^29,32^, whereas *Tenm2* and *Tenm4* are broadly expressed in most neurons, again making it difficult to envision a mechanism by which *Tenm2* and *Tenm4* that are expressed in nearly all neurons could encode specificity of a subset of synaptic connections. Finally, in CA1 neurons the postsynaptic deletion of latrophilins (the only known high-affinity ligands of teneurins) caused a loss of synapses, which would only be rescued by a latrophilin when that latrophilin contained the teneurin-binding site, suggesting that heterophilic teneurin-latrophilin interactions mediate synapse formation^35^.

Thus, the prevailing hypothesis that teneurins function in the development of synaptic connections via homophilic interactions is at present still unvalidated. Answers to two key questions that directly test this hypothesis are missing. First, are teneurins synaptic proteins, i.e., do they localize to the synaptic cleft, or are the phenotypes of impaired synaptic connections after teneurin deletions due to other neuronal functions of teneurins, such as in neuronal migration or axonal pathfinding? No high-resolution localization of a teneurin in brain is available to address this question. Second, do teneurins act in building synaptic connections as both pre- and postsynaptic proteins, or only as either pre- or postsynaptic proteins? All conclusions suggesting a role for homophilic interactions of teneurins in synaptic connections were based on correlations of their expression patterns in pre- and postsynaptic neurons or on imaging of axons, and all of these conclusions were tested with global deletions of teneurins in both pre- and postsynaptic neurons without selective pre- or postsynaptic manipulations (see references cited above). Only one study^29^ examined pre- vs. postsynaptic manipulations, but this study mapped the trajectory of labeled axons in hippocampal sections as a function of *Tenm3* deletions. From this study, it remained unclear whether the changes observed with both pre- and postsynaptic deletions reflect alterations in synaptic connections, changes in axonal pathfinding, or epiphenomena induced by small shifts in the plane of sectioning^29^. Given the central functions of teneurins in brain^25–30^, these are important questions that have implications for our understanding of how neural circuits are wired.

The present study aims to address these two key questions and to determine whether teneurins act presynaptically or postsynaptically or both in synapse formation. Our results show that *Tenm3* protein is localized to the synaptic cleft, where it forms nanoclusters in presynaptic active zones. Moreover, our data document that pre- but not postsynaptic deletions of *Tenm3* and *Tenm4* produce robust impairments in synaptic connectivity. Thus*Tenm3* and *Tenm4* function in brain as presynaptic adhesion molecules that are essential for the formation and/or maintenance of at least a subset of excitatory synapses by binding to postsynaptic non-teneurin ligands since postsynaptic teneurins are not required.

## Results

### Synaptic projections from the medial entorhinal cortex (MEC) to the proximal CA1 region and distal subiculum contain Tenm3

**Fig. 1:**
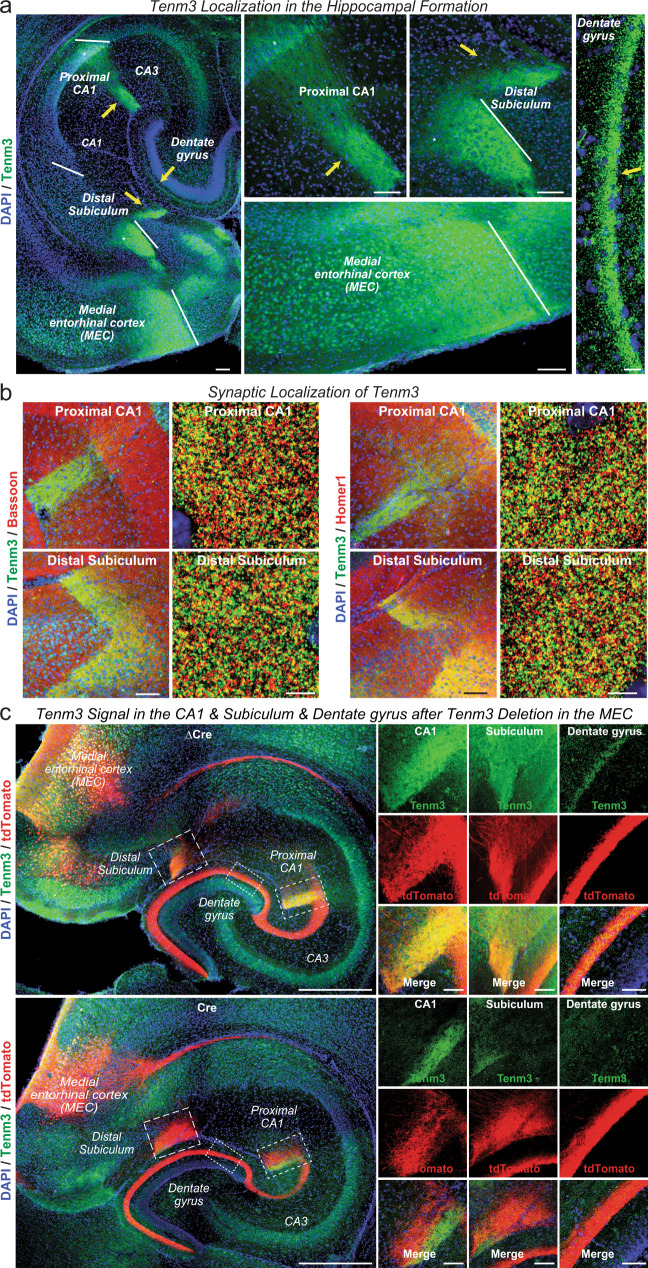
Teneurin-3 (Tenm3) is a synaptic protein that is present in discrete areas of the hippocampal formation. **a** Overall localization of Tenm3 in the hippocampal formation. Horizontal brain sections from a juvenile wild-type mouse were stained with antibodies to Tenm3 (green) and DAPI (blue). Left panel, overview; right four panels, higher magnification views of the proximal CA1, distal subiculum, medial entorhinal cortex (MEC), and dentate gyrus as indicated. Arrows point to areas of intense labeling, such as the S. lacunosum-moleculare of the proximal CA1, the molecular layer of distal subiculum, and the dentate gyrus. Asterisks represent the cell body of both proximal CA1 and distal subiculum. **b** Tenm3 is co-localized with excitatory synapse markers in the S. lacunosum-moleculare of the proximal CA1 and the molecular layer of distal subiculum. Panels show representative medium- and high-resolution images of sections from the proximal CA1 (top panels) and the distal subiculum (bottom panels) that were double-labeled with antibodies to Tenm3 (green) and Bassoon (red), a presynaptic active zone marker (left panels) or Tenm3 (green) and Homer1 (red), a postsynaptic scaffolding protein (right panels). For additional experiments and quantifications, see Supplementary Fig. 1a–d. **c** Tenm3 deletion in the MEC causes loss of Tenm3 staining in hippocampal target areas of MEC neurons. Left, representative images of horizontal sections from Tenm3 cKO mice whose MEC was stereotactically infected with AAVs co-expressing tdTomato (red) with either mutant Cre (ΔCre, as a control, top left panel) or Cre (bottom left panel); sections were stained for Tenm3 (green) and DAPI (blue). Right, representative high-resolution views of the proximal CA1 and distal subiculum taken from the experiment shown in the left panels. Note that the Tenm3 deletion in the MEC abolishes nearly all overlap of tdTomato signal derived from the MEC with the local Tenm3 signal in the CA1 region and subiculum. Scale bars: 0.1 mm (**a**, left and middle panels), 10 µm (**a**, right panel), 100 µm (**b**, left panel in each group), 5 µm (**b**, right panel in each group), 0.5 mm (**c**, left panel) and 50 µm (**c**, right panel).

Using a previously characterized Tenm3 antibody^29^ for immunocytochemistry of horizontal brain sections, we found that Tenm3 was enriched in the MEC and in discrete subcompartments of the hippocampus: The S. lacunosum-moleculare of a small part of the proximal CA1 region, the molecular layer of the distal subiculum, and the molecular layer of the dentate gyrus (Fig. 1a). The Tenm3 staining pattern we identified is consistent with a synaptic localization because the stained subcompartments of the hippocampus are enriched in synapses. The S. lacunosum-moleculare of the CA1 and the molecular layer of the subiculum are the sites of dense input synapses derived from MEC neurons^37–40^. Double-labeling of hippocampal sections for Tenm3 and for markers of all presynaptic terminals (Bassoon), of excitatory (vGluT1) or of inhibitory presynaptic terminals (vGAT), or of postsynaptic excitatory specializations (Homer1) indicated that Tenm3 is co-localized with excitatory but not inhibitory synapse markers (Fig. 1b, and Supplementary Fig. 1a–d). As observed previously^29^, the Tenm3-positive brain regions are synaptically connected, which was proposed to suggest that Tenm3 expression in pre- and the postsynaptic neurons of the MEC→CA1 region and MEC→subiculum circuits could serve to establish the excitatory synapses of these circuits via a homophilic interaction.

To test whether Tenm3 is targeted to presynaptic terminals of MEC→CA1 region and MEC→subiculum synapses, we deleted *Tenm3* from the MEC in conditional KO (cKO) mice and examined the Tenm3 signal as a function of the MEC deletion (Supplementary Fig. 1e). We stereotactically infected the MEC of newborn *Tenm3* cKO mice with AAVs that co-express tdTomato and active (Cre) or mutant inactive Cre-recombinase (ΔCre, as a control)^41^. We then examined the presence of tdTomato and Tenm3 in various parts of the hippocampus as a function of the *Tenm3* deletion in the MEC at postnatal day 13 (P13), a time point when most synaptogenesis in the hippocampus is completed.

Confocal microscopy revealed that under control conditions (i.e., when the MEC was infected with AAVs expressing inactive ΔCre), the AAV-derived tdTomato signal largely overlapped with the Tenm3 signal in the synaptic layers of the proximal CA1 region and distal subiculum (Fig. 1c). However, expression of Cre in the MEC suppressed the Tenm3 signal in tdTomato-positive areas of the proximal CA1 region and distal subiculum, suggesting that presynaptic projections from the MEC contain Tenm3. Depending on the precise extent of the MEC infection with Cre- and tdTomato-expressing AAVs, some Tenm3 signal remained, but the Tenm3 signal was then segregated from the tdTomato signal (Fig. 1c). These results show that presynaptic inputs from the MEC contribute most of the Tenm3 staining in the CA1 region and subiculum, but do not exclude a partially postsynaptic localization. Note that these experiments also revealed, as expected, that expression of Cre in MEC neurons produces tdTomato-positive projections to the dentate gyrus that, however, do not contain Tenm3 (Fig. 1c).

### Tenm3 forms presynaptic nanoclusters in excitatory hippocampal synapses

**Fig. 2:**
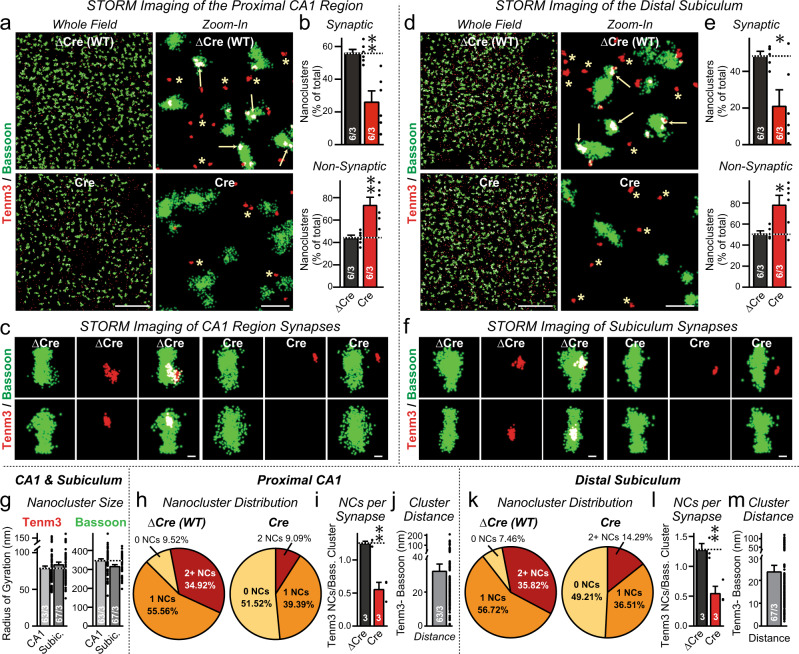
Super-resolution STORM imaging reveals synaptic Tenm3 nanoclusters in the proximal CA1 and the distal subiculum that are adjacent to presynaptic active zones visualized with antibodies to Bassoon. **a** Representative images of Tenm3 nanoclusters (red) and presynaptic Bassoon macroclusters (green) show that the majority of Tenm3 nanoclusters in the proximal CA1 region are localized close to presynaptic active zones. Left images show overviews; right images, zoom-ins, in the whole field (left) and zoon-in field (right) at proximal CA1. Mice were infected with AAVs at P0 and analyzed at P13 (arrows = synaptic Tenm3 nanoclusters co-localized with Bassoon macroclusters; asterisks = non-synaptic Tenm3 nanoclusters that are >80 nm separated from presynaptic Bassoon macroclusters). **b** The Tenm3 deletion suppresses synaptic Tenm3 nanoclusters in the proximal CA1 region. Summary graphs show the percentage of Tenm3 nanoclusters that are synaptic (top) or non-synaptic (bottom) in control-infected (black) or Cre-expressing AAVs Tenm3 cKO mice (red). **c** Representative images of individual synapses in the proximal CA1 containing Tenm3 nanoclusters in mice without (ΔCre, left) and with deletion of Tenm3 in the MEC (Cre, right). **d**–**f** Same as **a**–**c**, except that brain sections were analyzed from distal subiculum. **g** Tenm3 nanoclusters occupy approximately 6% of the presynaptic active zone. Summary graphs depict the radius of Tenm3 nanoclusters (left) and Bassoon macroclusters (right) in the proximal CA1 and distal subiculum. **h** Most (>90%) presynaptic active zones visualized as Bassoon macroclusters contain 1–2 Tenm3 nanoclusters in WT synapses in the proximal CA1 region. **i** Summary graph of the average number of nanoclusters per Bassoon-positive excitatory synapse in Tenm3 WT and neuron cKO from proximal CA1. **j** The minimum intercluster distance between Bassoon and Tenm3 clusters. **k**–**l** Same as **i**, **j**, except that brain sections were analyzed from distal subiculum. Bar graphs show means ± SEM; numbers of sections/mice are indicated in the bars. Statistical significance was assessed using two-sided Student’s t-test (*p < 0.05; **p < 0.01). For additional experiments and quantifications, see Supplementary Fig. 2. Scale bars: 10 µm (**a**, **d**, left panel), 1 µm (**a**, **d**, right panel) and 200 nm (**c**, **f**).

**Fig. 3:**
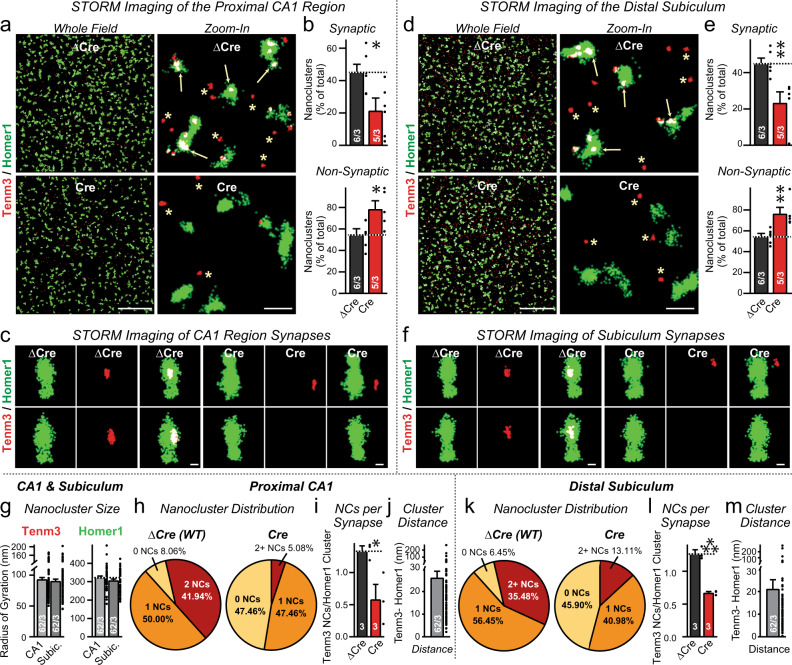
Super-resolution STORM imaging uncovers Tenm3 nanoclusters adjacent to postsynaptic densities that are visualized using staining for the postsynaptic marker Homer1. **a** Representative images of Tenm3 nanoclusters (red) and Homer1 (green) visualized using direct stochastic optical reconstruction microscopy in the whole field (left) and zoon-in field (right) at proximal CA1. Brain sections stained include Tenm3 WT (top) and neuron cKO (bottom) at P13, showing multiple Homer1-positive synapses contain Tenm3 nanoclusters. Arrows indicate Tenm3 nanoclusters overlap with Homer1-positive synapses (synaptic Tenm3 nanoclusters). Asterisks represent Tenm3 nanoclusters that are not close to Homer1-positive synapses (non-synaptic Tenm3 nanoclusters). **b** Summary graph of the percent of Tenm3 nanoclusters close to Homer1 macroclusters from the whole field imaging in Tenm3 WT (top) and neuron cKO (bottom) brain sections. The percent of synaptic Tenm3 nanoclusters was decreased in neuron cKO brain sections in proximal CA1. **c** Representative images of individual synapses from proximal CA1 with Tenm3 WT (left) and neuron cKO (right) showing Homer1-positive synapses with Tenm3 nanoclusters. **d**–**f** Same as **a**–**c**, except that brain sections were analyzed from distal subiculum. The percent of synaptic Tenm3 nanoclusters was decreased in neuron cKO brain sections in distal subiculum. **g** Tenm3 nanoclusters and Homer1 macroclusters size measured as the radius of gyration. **h** Pie charts showing the distribution of Homer1-positive excitatory synapse without or with Tenm3 nanoclusters (i.e., 1 and 2+) in Tenm3 WT (left) and neuron cKO (right) in proximal CA1. **i** Summary graph of the average number of nanoclusters per Homer1-positive excitatory synapse in Tenm3 WT and neuron cKO from proximal CA1. **j**. The minimum intercluster distance between Homer1 and Tenm3 clusters. **k**–**m** Same as **h**–**j**, except that brain sections were analyzed from distal subiculum. Bar graphs show means ± SEM; numbers of sections/mice are indicated in the bars. Statistical significance was assessed using two-sided Student’s t-test (*p < 0.05; **p < 0.01). For additional experiments and quantifications, see Supplementary Fig. 2. Scale bars: 10 µm (**a**, **d**, left panel), 1 µm (**a**, **d**, right panel) and 200 nm (**c**, **f**).

Because of the resolution limits of confocal microscopy, the immunolocalization results only indicate that Tenm3 is localized, at least in part, to presynaptic terminals, but do not reveal if Tenm3 is actually present in synaptic junctions. Therefore we used STORM super-resolution microscopy to characterize the precise localization of Tenm3^42–47^. We analyzed the localization of Tenm3 in the proximal CA1 region and the distal subiculum in relation to that of the presynaptic active zone protein Bassoon (Fig. 2) and of the postsynaptic density protein Homer1 (Fig. 3), using staining of the sections with a non-specific antibody as an additional control (Supplementary Fig. 2). In these experiments, we infected the ipsilateral CA1 region, subiculum, and MEC of newborn *Tenm3* cKO mice with AAVs expressing mutant inactive (ΔCre) or active Cre-recombinase (Cre), and analyzed cryosections of the infected area at P13 by STORM.

STORM imaging revealed that Tenm3 was not diffusely distributed in the hippocampus, but formed discrete nanoclusters with a ~80 nm radius. In contrast, Bassoon and Homer1 were present in bigger macroclusters with a ~300 nm radius that corresponds to pre- and postsynaptic specializations, respectively (Figs. 2a, d, g, 3a, d, g;^42,47^). Approximately half of the Tenm3 nanoclusters in the proximal CA1 region and the distal subiculum were in contact with Bassoon and Homer1 macroclusters (i.e., were synaptic), whereas the remaining Tenm3 nanoclusters were localized close to, but not overlapping with, Bassoon or Homer1 macroclusters (i.e., may at least be partly non-synaptic; Figs. 2b, e, 3b, e and Supplementary Fig. 2b, e). Synaptic Tenm3 nanoclusters were separated by ~20–30 nm from both Bassoon and Homer1 macroclusters, suggesting that the Tenm3 nanoclusters are localized in the synaptic cleft (Fig. 2g, h, Supplementary Fig. 2c, f–h). Under control conditions, the vast majority of Bassoon and Homer1 macroclusters contained at least one Tenm3 nanocluster (average ~1.3 nanoclusters/synapse; Figs. 2c, h–l, 3c, h–l). Deletion of *Tenm3* in the MEC, CA1 region, and subiculum greatly decreased (>2-fold) the proportion of Tenm3 nanoclusters in synaptic specializations, whereas the proportion of non-synaptic Tenm3 nanoclusters showed a relative increase (Figs. 2b, e, 3b, e). Moreover, deletion of *Tenm3* massively lowered (>2-fold) the average number of Tenm3 nanoclusters per Bassoon and Homer1 macroclusters (Figs. 2j, l, m, 3j, l, m).

The STORM results demonstrate that Tenm3 is an intrinsic component of nearly all synaptic junctions in the proximal CA1 region and distal subiculum. However, Tenm3 is not exclusively localized to synapses, but appears to be also present outside of synapses, similar to neurexins that also form presynaptic nanoclusters^47^. It is possible that the apparently extrasynaptic Tenm3 nanoclusters are expressed in glia or in interneurons. Moreover, the regional deletion of *Tenm3* did not ablate all synaptic Tenm3 nanoclusters, which might be due to the expression of Tenm3 in presynaptic projections from other brain regions such as the contralateral hippocampus, or to an incomplete deletion of Tenm3 in the infected brain regions.

### Strategies for the pre- and postsynaptic inactivation of Tenm3 and Tenm4

The confocal and STORM imaging results localize Tenm3, at least in part, to the synaptic cleft, but do not inform us on the function of synaptic Tenm3, nor do they reveal whether Tenm3 (and by extension, other teneurins) function pre- and/or postsynaptically. At this point, therefore, it is important to determine whether teneurins are essential for synapse assembly and whether they function pre- and/or postsynaptically. However, the restricted expression pattern of Tenm3 (Fig. 1) indicates that Tenm3 is present in only a subset of hippocampal synapses whose separate functions may be difficult to monitor. In contrast, single-cell RNA-seq data reveal that *Tenm2* and *Tenm4* are broadly expressed in most excitatory neurons, and that neurons co-express multiple teneurins^48^, raising the possibility of different teneurins are functionally redundant (Supplementary Fig. 3). Since especially *Tenm3* and *Tenm4* are co-expressed in many hippocampal neurons and conditional KO (cKO) mice are available for these teneurins, we probed the functional effects of deleting both *Tenm3* and *Tenm4* in the hippocampal formation using double conditional KO (DcKO) mice. In these experiments, we focused on the MEC and on the CA1 region of the hippocampus because *Tenm3* and *Tenm4* are expressed in these brain regions at least partially with the SSB- splice variant that binds to latrophilins (Supplementary Fig. 4).

**Fig. 4:**
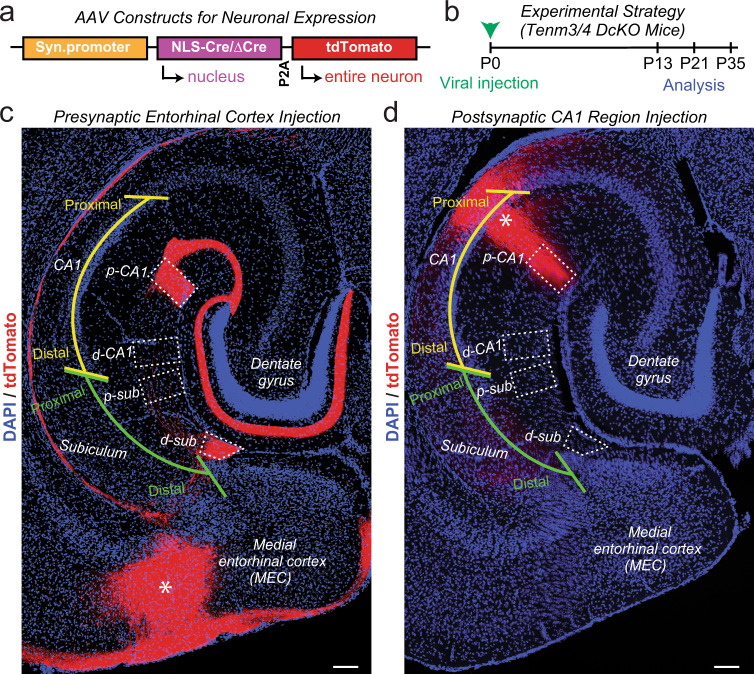
Strategy for selective deletions of both Tenm3 and Tenm4 from the MEC or CA1 in Tenm3/4 double cKO mice to achieve exclusively pre- or postsynaptic ablation of Tenm3 and Tenm4 expression. **a** Design of AAVs used for Tenm3/4 deletions. The synapsin promoter drives bicistronic expression of Cre or ∆Cre (control) and soluble tdTomato, enabling visualization of infected neurons and their axonal projections. **b** Experimental approach. Tenm3/4 DcKO mice were stereotactically infected with AAVs at P0, and analyzed at P13, P21, or P35. **c** Representative image of a horizontal section of the hippocampal formation of a control mouse at P13 whose MEC was infected with ΔCre- and tdTomato-expressing AAVs at P0 (asterisk = infected area). Note that MEC neurons project abundant tdTomato-positive axons to the S. lacunosum-moleculare of the proximal CA1 (p-CA1) and the molecular layer of distal subiculum (d-sub), but not to the distal CA1 (d-CA1) or proximal subiculum (p-sub). Dotted boxes indicate areas that were quantitatively analyzed in Fig. 5a–c, Supplementary Figs. 4, 6. **d** Same as **c**, except that the CA1 region was infected stereotactically with ΔCre- and tdTomato-expressing AAVs. Note that the CA1 region neurons, as visualized in horizontal sections, send sparse axons to distal subiculum. Dotted boxes indicate areas that were quantitatively analyzed in Fig. 5g–i, Supplementary Figs. 5, 7. Scale bars: 0.5 mm (**c**, **d**).

To selectively inactivate *Tenm3* and *Tenm4* presynaptically, we infected the MEC of *Tenm3* and *Tenm4* DcKO mice with AAVs that co-express tdTomato with ΔCre (control) or Cre (test). We then examined MEC→CA1 region and MEC→subiculum synapses, and in a subset of experiments also studied MEC→dentate gyrus synapses (Fig. 4a). To selectively delete *Tenm3* and *Tenm4* postsynaptically, conversely, we infected the CA1 region and analyzed MEC→CA1 region synapses. We used stereotactic manipulations to infect the MEC or CA1 region in newborn mice before most synaptogenesis occurred, and analyzed synaptic connections in these mice at postnatal days P13, P21, and P35 after synaptogenesis (Fig. 4b). Horizontal sections of the hippocampus illustrated that AAV injections into the neonatal MEC and the proximal CA1 region enable precise mapping of infected neurons and their projections via co-expressed tdTomato (Fig. 4c, d).

### Tenm3/4 deletions in the MEC but not in the CA1 decrease the overall excitatory synapse number in the hippocampus

**Fig. 5:**
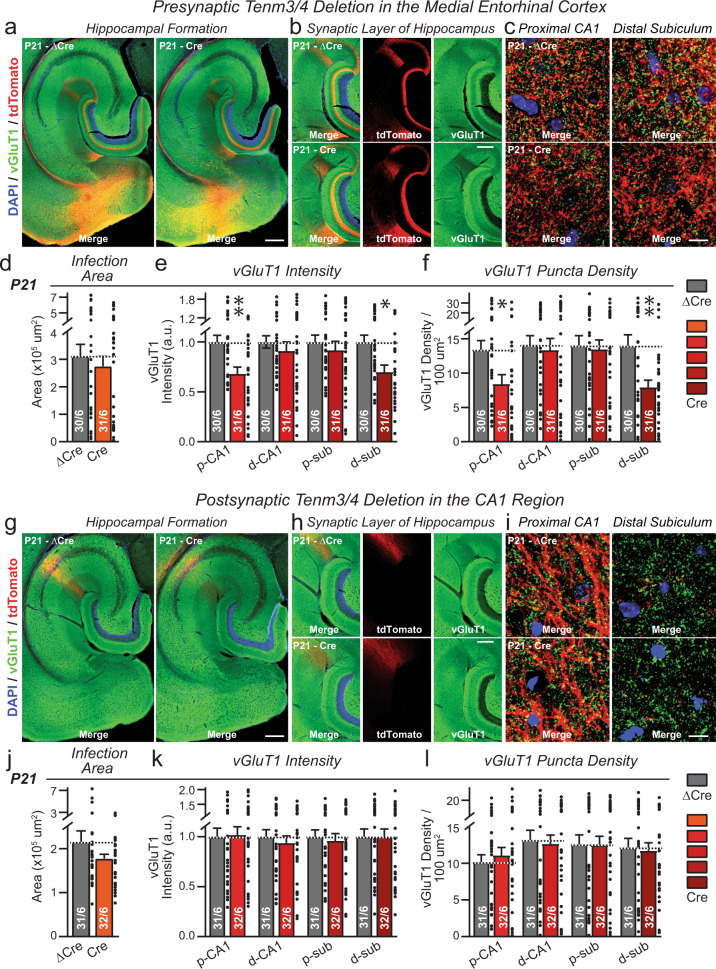
Presynaptic deletion of *Tenm3* and *Tenm4* in the MEC decreases the overall excitatory synapse density in the proximal CA1 and distal subiculum, whereas postsynaptic deletion of *Tenm3* and *Tenm4* in the proximal CA1 has no effect on synapse density. **a** Representative overviews of the excitatory synapse density in the hippocampal formation from Tenm3/4 DcKO mice at P21 after presynaptic expression of ΔCre (control) or Cre in the MEC at P0. Horizontal sections were stained for the excitatory synaptic marker (vGluT1, green) and for DAPI (blue) at P21. **b** Representative images of the hippocampus proper from the experiment shown in (**a**). **c** Representative higher resolution images of vGluT1 staining in the proximal CA1 region (left) and distal subiculum region (right). Quantification of the infected MEC area shows that the extent of viral infection was similar between control (∆Cre) and test mice (Cre) mice. For the P13 and P35 time points, see Supplementary Fig. 4. **e**, **f** Presynaptic deletion of Tenm3/4 in MEC neurons significantly decreases the overall density of excitatory synapses in the areas to which the MEC neurons project as revealed by their tdTomato signal, namely the proximal CA1 (p-CA1) and the distal subiculum (d-sub). The distal CA1 (d-CA1) and proximal subiculum (p-sub), which are lacking tdTomato-positive MEC projections, exhibited no change in synapse density. Summary graphs depict quantifications of the global vGluT1 staining intensity (**e**) and the vGlut1-positive puncta density (**f**) as two measures of synapse density. **g**–**l** Same as **a**–**f**, except that the postsynaptic proximal CA1 was infected with ΔCre- and Cre-expressing AAVs. The extent of viral infection was not different between control (∆Cre) and test (Cre) mice. Postsynaptic deletion of Tenm3/4 in proximal CA1 had no effect on the density of vGluT1-positive synapses. Bar graphs show means ± SEM; numbers of sections/mice are indicated in the bars. Statistical significance was assessed using two-sided Student’s t-test and Two-way ANOVA (*p < 0.05; **p < 0.01). For comparable data at the P13 and P35 time points, see Supplementary Figs. 5–8. Scale bars: 1 mm (**a**, **g**), 0.2 mm (**b**, **h**) and 10 µm (**c**, **i**).

We first studied the effect of pre- or postsynaptic deletions of *Tenm3* and *Tenm4* on the overall synapse density in the hippocampus (Fig. 5 and Supplementary Figs. 5–8). In these experiments, we measured the overall intensity and density of excitatory and inhibitory synapses by immunocytochemistry for vGluT1 and vGAT, respectively.

Expression of Cre in the MEC of *Tenm3* and *Tenm4* DcKO mice decreased the intensity and density of vGluT1-positive synaptic puncta in the proximal CA1 region and the distal subiculum, but not in the distal CA1 region or proximal subiculum (Fig. 5a–f and Supplementary Fig. 5). The decrease in synapse density was observed as a non-significant trend at P13, and became robustly significant (~30–40% decrease) at P21 and P35. No changes in the intensity or density of vGAT-positive inhibitory synaptic puncta were detected (Supplementary Fig. 7). Thus, presynaptic deletions of *Tenm3* and *Tenm4* in MEC neurons cause a partial loss of synapses in their target areas that is selective to these target areas. Since these target areas (the proximal CA1 and distal subiculum) also receive inputs from many other brain regions, is not surprising that only a subset of synapses was lost.

In contrast to the presynaptic *Tenm3/4* deletions, postsynaptic *Tenm3/4* deletions by expression of Cre in the proximal CA1 had no effect on the synapse intensity and density in the CA1 region at any time point (Fig. 5g–l and Supplementary Fig. 6). The intensity and density of vGAT-positive synaptic puncta were also not changed (Supplementary Fig. 8). These results indicate that postsynaptic Tenm3 and Tenm4 are not essential for the formation and maintenance of input synapses in CA1 region neurons.

### SynaptoTag tracing demonstrates a presynaptic requirement for teneurins in synapse formation

**Fig. 6:**
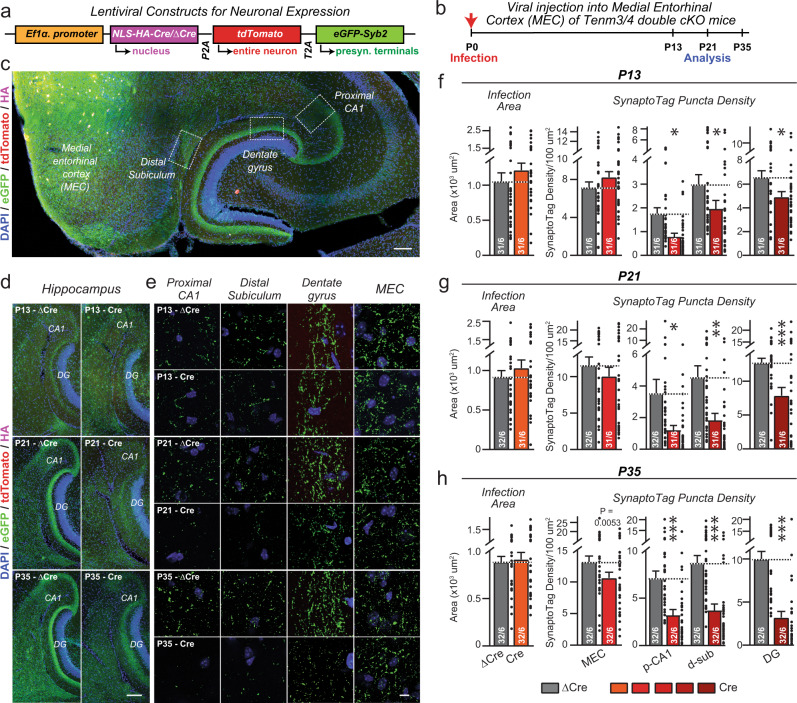
SynaptoTag mapping reveals that inactivation of Tenm3 and Tenm4 expression in the MEC severely impairs synapse formation by entorhinal cortex projections onto neurons in the proximal CA1, distal subiculum, and dentate gyrus. **a** Design of SynaptoTag lentiviruses used for the presynaptic tracing of synaptic connections. The Ef1α promoter drives the expression of HA-tagged Cre or ∆Cre, tdTomato, and a presynaptic eGFP-synaptobrevin-2 fusion protein (eGFP-Syb2). The HA signal visualizes the viral injection area, the soluble tdTomato signal visualizes axonal projections, whereas the eGFP-Syb2 signal visualizes presynaptic specializations. Sections were stained for HA (magenta), tdTomato (red), eGFP (green) and DAPI (blue). **b** Experimental strategy. The MEC neurons of Tenm3/4 DcKO mice were stereotactically injected with the SynaptoTag lentiviruses at P0, and mice were analyzed by imaging at P13, P21, and P35. **c** Representative image of a horizontal section of the hippocampal formation from a SynaptoTag experiment. Boxes illustrate the areas that were analyzed by eGFP-Syb2 signal quantification. Representative low (**d**) and higher resolution images (**e**) at P13 (top), P21 (middle), or P35 (bottom) of the SynaptoTag signals in hippocampal sections after infection of the MEC with control (∆Cre, left) and test SynaptoTag lentiviruses (Cre, right). The panels in **d** show overviews, while the panels in **e** depict the indicated areas of the hippocampal formation. **f**–**h** Quantifications of the SynaptoTag signal reveal that the Tenm3/4 deletion in the MEC causes a large decrease in MEC-derived synapses in the proximal CA1, distal subiculum, and dentate gyrus, but not in the MEC itself, at all time points analyzed (**f**, P13; **g**, P21; **h**, P35). Left graphs show the size of the lentivirally infected areas in the MEC; right graphs show the SynaptoTag puncta density in the MEC, proximal CA1 (p-CA1), distal subiculum (d-sub), and dentate gyrus (DG). Data are means ± SEM; numbers of sections/mice are indicated in the bars. Statistical significance was assessed using two-sided Student’s t-test and Two-way ANOVA (*p < 0.05; **p < 0.01; ***p < 0.001). For additional experiments and quantifications, see Supplementary Fig. 9. Scale bars: 0.5 mm (**c**), 0.2 mm (**d**) and 20 µm (**e**).

To independently validate the conclusion of the overall synapse density measurements that presynaptic Tenm3/4 are required for establishing synapses, we used SynaptoTag, a tool that specifically monitors assembly of presynaptic specializations^49^. We infected the MEC of *Tenm3/4* DcKO mice with a lentivirus that co-expressed Cre or ΔCre (control) with both tdTomato (to trace axons) and EGFP-tagged synaptobrevin-2 (to mark presynaptic terminals) (Fig. 6a). Again, stereotactic infections were performed at P0, and the brains were analyzed at P13, P21, and P35 (Fig. 6b). Overview sections of the hippocampal area after SynaptoTag infections in the MEC confirmed that the MEC projects broadly to multiple regions (Fig. 6c), and zoom-ins of target regions uncovered the presence of EGFP-positive synaptic puncta in these areas (Fig. 6d and Supplementary Fig. 9a–d).

Quantitative analyses demonstrated that at all time points, the deletion of *Tenm3* and *Tenm4* caused a significant decrease in the density, size, and intensity of SynaptoTag puncta in the proximal CA1, the distal subiculum, and the dentate gyrus (Fig. 6d–h and Supplementary Fig. 9). The effect was most pronounced at P35, when the *Tenm3/4* deletion induced a large decrease (>50%) in synapse numbers as measured via the SynaptoTag signal. Quantifications of the infected area in the MEC ensured that the extent of the original infection was similar between the Cre viruses and the ΔCre control viruses (Fig. 6f–h). These data confirm that presynaptic expression of *Tenm3* and *Tenm4* in MEC neurons is essential for the establishment of synaptic connections of these neurons in the hippocampal region.

### Retrograde rabies virus tracing confirms that postsynaptic *Tenm3* and *Tenm4* are dispensable for synaptic connectivity

In the experiments demonstrating that postsynaptic deletions of *Tenm3* and *Tenm4* have no effect on the overall synapse density, different from presynaptic deletions (Figs. 4, 5 and Supplementary Figs. 5–8), changes in synapse numbers induced by postsynaptic Tenm3/4 deletions may have been overlooked owing to the high synapse density in the CA1 region of the hippocampus. To independently test the possible role of postsynaptic teneurins in synaptic connectivity, we used retrograde rabies virus tracing, the ‘gold standard’ for monitoring synaptic connectivity^35,50^.

**Fig. 7:**
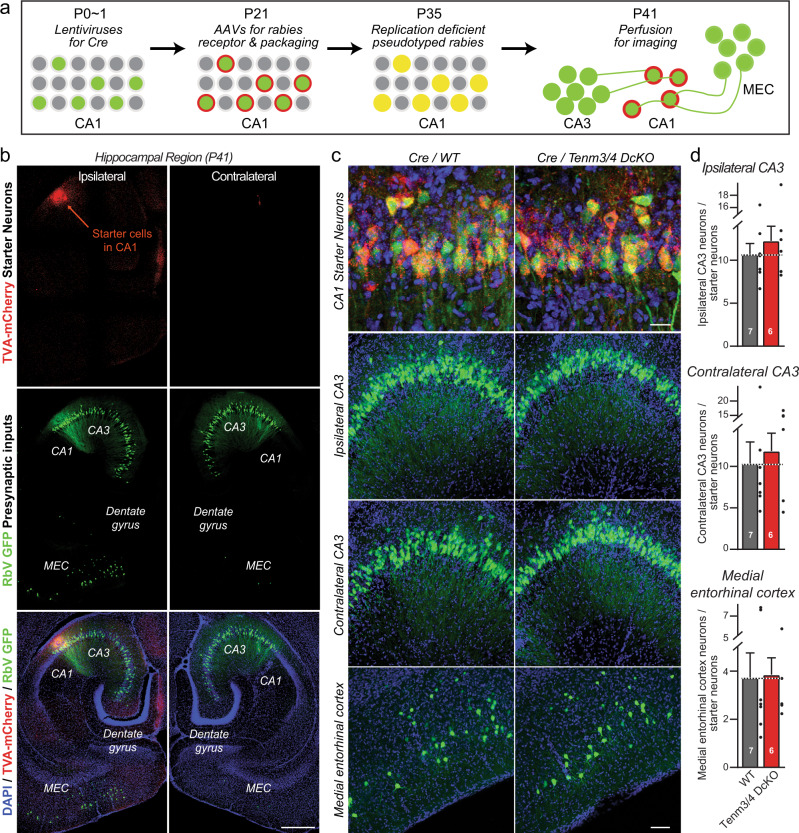
Retrograde trans-synaptic pseudorabies virus tracing demonstrates that the postsynaptic deletion of Tenm3 and Tenm4 in CA1 region neurons has no effect on the formation of synapses on CA1 neurons. **a** Experimental strategy. The proximal CA1 neurons of WT or Tenm3/4 DcKO mice were sparsely infected unilaterally with lentiviruses encoding Cre-recombinase at P0. The same proximal CA1 neurons were then superinfected with AAVs encoding the Cre-dependent mCherry-tagged pseudorabies virus receptor and pseudorabies virus packaging proteins at P21, which are therefore only expressed in neurons containing Cre. Finally, the proximal CA1 was infected with pseudorabies viruses encoding eGFP at P35, and the ipsi- and contralateral hippocampal CA3 and the ipsilateral medial entorhinal cortex were analyzed by imaging at P41. Postsynaptic ‘starter’ neurons in the tracing experiments are double-positive for both mCherry and eGFP, whereas presynaptic input neurons are positive only for eGFP. **b** Representative images of a pseudorabies virus tracing experiment in the hippocampal formation (red, starter neurons expressing the mCherry-tagged pseudorabies virus receptor; green, eGFP-positive neurons containing pseudorabies viruses). **c** Representative higher resolution images of retrograde trans-synaptic tracing experiments in control mice (left images) and mice with a postsynaptic deletion of Tenm3/4 (right images). Images show starter neurons (top), ipsi- and contralateral presynaptic CA3 region input neurons (middle rows), and ipsilateral MEC input neurons (bottom). **d** Quantifications of presynaptic inputs onto postsynaptic proximal CA1 neurons as a function of the postsynaptic Tenm3/4 deletions show that postsynaptic Tenm3 and Tenm4 are not required for normal synaptic connections formed by MEC and CA3 neurons onto CA1 neurons. Data are means ± SEM; numbers of mice are indicated in the bars. Statistical significance was assessed using two-sided Student’s t-test (non significant comparisons are not indicated). Scale bars: 0.5 mm (**b**), 20 µm (**c**, top panel) and 100 µm (**c**, bottom three panels).

We sparsely infected CA1 region neurons of newborn *Tenm3*/*4* DcKO and control mice with lentiviruses encoding Cre, re-infected the same CA1 region at P21 with AAVs encoding the Cre-dependent receptor and packaging components for pseudotyped rabies viruses, and finally infected the same CA1 region neurons again at P35 with pseudotyped rabies virus (Fig. 7a). In this manner*Tenm3* and *Tenm4* were deleted in postsynaptic CA1 neurons before most synaptic connections are formed. We analyzed the mice at P41 by quantifying the trans-synaptic spread of postsynaptically produced pseudotyped rabies viruses in cryosections, based on the expression of EGFP by retrogradely trans-synaptically transported viruses. The results demonstrate that postsynaptic deletion of *Tenm3* and *Tenm4* in CA1 region neurons has no effect on the extent of synaptic connections formed by presynaptic inputs onto these neurons (Fig. 7b–d).

### Electrophysiological recordings document that postsynaptic Tenm3/4 deletions have no effect on MEC→CA1 or CA3→CA1 synapses

It is possible that postsynaptic Tenm3/4 deletions have no effect on synapse numbers but alter synaptic transmission, even though presynaptic Tenm3/4 deletions decrease synapse numbers. To probe for functional changes induced by postsynaptic Tenm3/4 deletions, we performed electrophysiological recordings from CA1 pyramidal neurons in acute slices. Acute slices were cut from Tenm3/4 DcKO mice at P19-22 after the CA1 region of these mice had been infected at P0 with AAVs encoding ΔCre (control) or Cre (Supplementary Fig. 10a). As with the other experiments of the present study, this strategy thus examines the effect of Tenm3/4 deletions on synaptic connectivity for the initial development as well as the maintenance of synaptic connections.

**Fig. 8:**
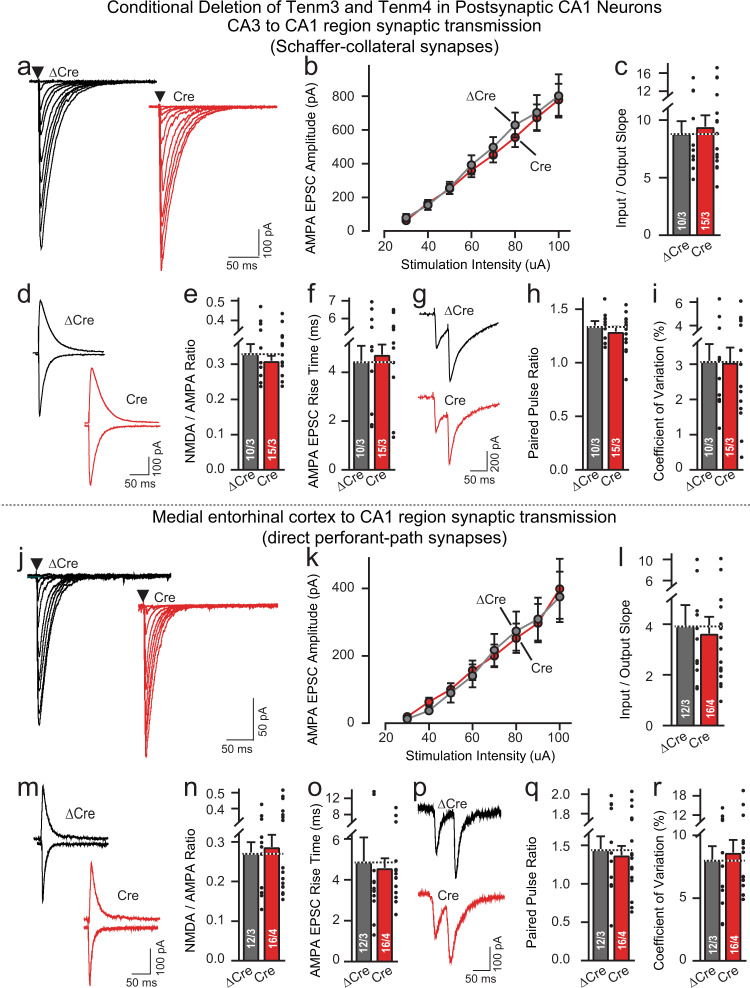
Postsynaptic deletion of Tenm3 and Tenm4 in CA1 region neurons has no effect on MEC→CA1 or CA3→CA1 synaptic transmission. Data are from patch-clamp whole-cell recordings in acute hippocampal slices from Tenm3 and Tenm4 DcKO mice at P19-22. The proximal CA1 neurons of mice were infected unilaterally with AAVs encoding EGFP-tagged ∆Cre (control) or Cre at P0. Deletion of Tenm3 and Tenm4 in CA1 neurons has no effect on the synaptic responses mediated by CA3→CA1 Schaffer-collateral inputs (**a**, representative traces showing Schaffer-collateral EPSC at increasing stimulus; **b**, input-output curves; **c**, summary graph of the slopes of the input-output curves). The Tenm3/4 double deletion in CA1 neurons does not alter the AMPAR/NMDAR ratio and AMPA EPSC rise time in Schaffer-collateral inputs compared to ∆Cre controls (**d**, representative traces of AMPAR/NMDAR EPSC; **e**, summary graph of the AMPAR/NMDAR EPSC ratio; **f**, summary graph of the AMPA EPSC rise time). **g**–**i** Double deletion of Tenm3 and Tenm4 does not alter the paired-pulse ratios of Schaffer-collateral EPSC (**g**, representative traces; **h**, summary graph of the paired-pulse ratios), or the coefficient of variation (**i**, summary graph of the coefficient of variation). **j**–**r**, the same as **a**–**i**, except that the synaptic responses were analyzed by MEC→CA1 perforant-path inputs. Data are means ± SEM; numbers of sections/mice are indicated in the bars. Statistical significance was assessed using two-sided Student’s t-test (*p < 0.05; **p < 0.01; ***p < 0.001). For additional experiments, see Supplementary Fig. 10.

We measured synaptic transmission at both MEC→CA1 perforant-path synapses (Fig. 8j–r) and CA3→CA1 Schaffer-collateral synapses (Fig. 8a–i) by placing stimulating electrodes at their respective afferent axons (Supplementary Fig. 10c). However, we observed no change induced by the postsynaptic Tenm3/4 deletion in any parameter examined. Specifically, measurements of AMPA-receptor-mediated synaptic strength using input-output curves (to control for possible variations in stimulus strength) uncovered no difference between control synapses (Fig. 8a–c, j–l) and synapses lacking postsynaptic Tenm3/4, as did quantifications of the AMPA/NMDA ratio, the EPSC rise times, the paired-pulse ratios, or the coefficient of variation (Fig. 8d–i, m–r). Thus, the postsynaptic Tenm3/4 deletion has no major effects on the strength, glutamate receptor composition, kinetics, or release probability of MEC→CA1 perforant-path or CA3→CA1 Schaffer-collateral synapses.

### A new rabies virus tracing approach demonstrates that presynaptic teneurins are essential for MEC→CA1 synaptic connections

**Fig. 9:**
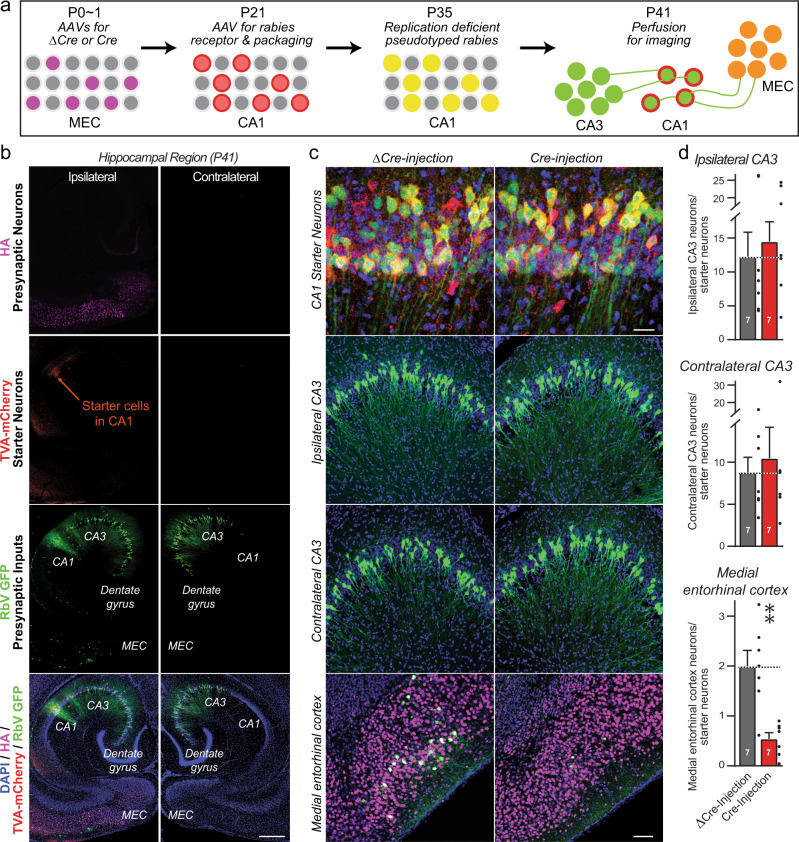
Pseudorabies tracing demonstrates that the presynaptic deletion of Tenm3 and Tenm4 in MEC neurons greatly decreases MEC→CA1 synaptic connectivity. **a** Experimental strategy that enables testing the effect of presynaptic Cre-mediated gene deletions on synaptic connections using retrograde pseudorabies virus tracing methods. MEC neurons of Tenm3/4 DcKO mice were infected unilaterally with AAVs encoding HA-tagged ∆Cre (control) or Cre at P0, proximal CA1 neurons were infected with AAVs encoding the Cre-independent mCherry-tagged rabies receptor and pseudorabies virus packaging proteins at P21. The same proximal CA1 neurons were infected with pseudorabies viruses encoding eGFP at P35, and the presynaptic inputs from the ipsi- and contralateral hippocampal CA3 region and the ipsilateral MEC were analyzed by imaging at P41. **b** Representative images of a pseudorabies virus retrograde tracing experiment analyzing the effect of a presynaptic Tenm3/4 deletion on synaptic inputs onto CA1 neurons (top, HA (magenta) signal in the MEC from ΔCre and Cre; second row, mCherry (red) signal in the CA1 from the pseudorabies virus receptor (starter cells); third row, eGFP (green) signal visualizing presynaptic inputs onto CA1 neurons; bottom row, merged images including a nuclear DAPI (blue) stain). **c** Representative higher resolution images of retrograde trans-synaptic tracing experiments in control mice (ΔCre, left images) and mice with a presynaptic deletion of Tenm3/4 in the MEC (right images). Images show starter neurons (top), ipsi- and contralateral presynaptic CA3 region input neurons (middle rows), and ipsilateral MEC input neurons (bottom). **d** Quantifications of presynaptic inputs onto postsynaptic proximal CA1 neurons as a function of the presynaptic Tenm3/4 deletion in the MEC show that presynaptic Tenm3 and Tenm4 in MEC are required for normal synaptic connections formed by MEC neurons onto CA1 neurons. Note that presynaptic Tenm3/4 deletions in the MEC do not affect synaptic connections formed by CA3 neurons onto CA1 neurons because the CA3 neurons were not manipulated genetically. Data are means ± SEM; numbers of mice are indicated in the bars. Statistical significance was assessed using two-sided Student’s t-test (**p < 0.01). Scale bars: 0.5 mm (**b**), 20 µm (**c**, top panel) and 100 µm (**c**, bottom three panels).

To validate these results that exclude a postsynaptic contribution of Tenm3 and Tenm4 to synapse formation on CA1 neurons, we sought to ascertain that the retrograde pseudotyped rabies virus tracing approach is in fact competent to measure synaptic connectivity. For this purpose, we tested the effect of the presynaptic *Tenm3* and *Tenm4* deletion on synaptic connectivity using a new variation of the pseudotyped rabies virus tracing approach that was applied above to examine the role of postsynaptic *Tenm3* and *Tenm4* in synaptic connectivity. Specifically, we infected the MEC of *Tem3/4* DcKO mice at P0 with AAVs expressing HA-tagged Cre or ΔCre (as a control), then infected the CA1 region of the same mice at P21 with AAVs expressing the mCherry-tagged receptor and packaging genes for pseudotyped rabies virus (but in Cre-independent versions), and finally infected the same CA1 region of these mice with pseudotyped rabies virus at P35 (Fig. 9a). We then analyzed the synaptic connectivity of the hippocampus at P41 by imaging postsynaptic pseudotyped rabies virus-infected neurons in the CA1 region that co-express mCherry with EGFP, presynaptic input neurons in the MEC, and the ipsi- and contralateral CA3 region that express EGFP without mCherry, and MEC neurons that were infected at P0 with Cre- or ΔCre-expressing AAVs that express HA-tagged Cre or ΔCre (Fig. 9b).

Quantifications revealed that the expression of Cre but not ΔCre in the MEC severely impaired (>75% decrease) MEC→CA1 region synaptic connections, confirming the requirement for presynaptic *Tenm3* and *Tenm4* at these synapses (Fig. 9c, d). In contrast, ipsi- and contralateral CA3 region→CA1 region synapses were unaffected, which is expected since the presynaptic CA3 region neurons were not infected with Cre-expressing viruses (Fig. 9c, d). Together, these data demonstrate that *Tenm3* and *Tenm4* are required presynaptically, but not postsynaptically, for MEC→CA1 region synapses.

### Presynaptic Tenm3/4 deletions in the MEC severely impair MEC→CA1 synaptic transmission measured electrophysiologically

**Fig. 10:**
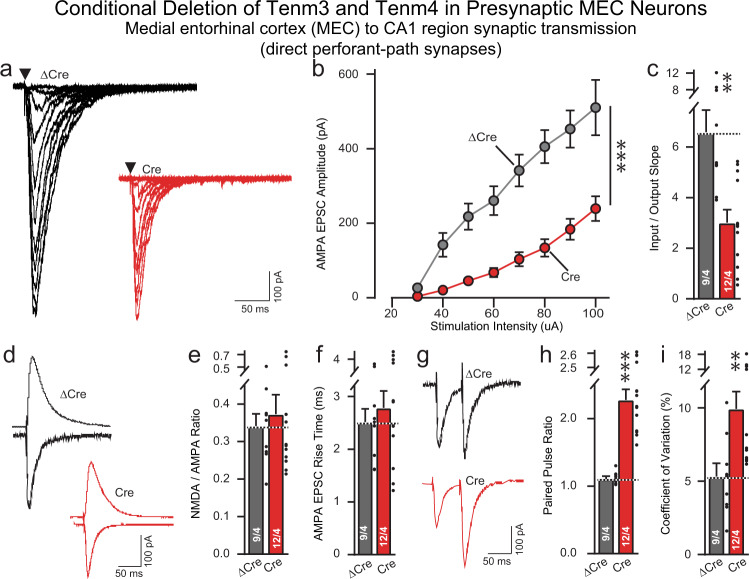
Presynaptic *Tenm3* and *Tenm4* deletions in the MEC severely impair MEC→CA1 synaptic transmission. Data are from patch-clamp whole-cell recordings in acute hippocampal slices from *Tenm3* and *Tenm4* DcKO mice at P19-22. The MEC neurons of mice were infected unilaterally with AAVs encoding EGFP-tagged ∆Cre (control) or Cre at P0. Presynaptic deletion in MEC neurons severely decreases perforant-path synaptic strength (**a** representative traces showing perforant-path EPSC at increasing stimulus; **b** input–output curves were used to control for differences in stimulus strength; **c** summary graph of the slopes of the input-output curves). The Tenm3/4 double deletion in CA1 neurons has no effect on the AMPAR/NMDAR ratio and AMPA EPSC rise time in perforant-path inputs compared to ∆Cre controls (**d**, representative traces of AMPAR/NMDAR EPSC; **e**, summary graph of the AMPAR/NMDAR EPSC ratio; **f**, summary graph of the AMPA EPSC rise time). Double deletion of Tenm3 and Tenm4 significantly increases the paired-pulse ratios of perforant-path EPSC (**g**, representative traces; **h**, summary graph of the paired-pulse ratios), or the coefficient of variation (**i**, summary graph of the coefficient of variation). Data are means ± SEM; numbers of sections/mice are indicated in the bars. Statistical significance was assessed using two-sided Student’s t-test (*p < 0.05; **p < 0.01; ***p < 0.001). For additional experiments, see Supplementary Fig. 10.

To confirm the SynaptoTag, synapse quantification, and rabies virus tracing results that presynaptic Tenm3/4 deletions impair synaptic connectivity, we recorded synaptic responses evoked in pyramidal CA1-region neurons by stimulation of perforant-path afferents in Tenm3/4 DcKO mice in which the MEC was infected with AAVs encoding either ΔCre (control) or Cre (Fig. 10; Supplementary Fig. 10b). Input-output curves revealed that the presynaptic Tenm3/4 deletion induced a dramatic suppression of synaptic strength that precisely matched the decrease in synaptic connectivity uncovered by rabies virus tracing (Fig. 10a–c). Analyses of the AMPA/NMDA ratio failed to detect a change, consistent with the notion that the remaining synapses after the Tenm3/4 deletion have no large changes in receptor composition (Fig. 10d). Moreover, the EPSC rise times were not altered (Fig. 10e). However, paired-pulse recordings and measurements of the coefficient-of-variation demonstrated large increases in both parameters, consistent with a decrease in release probability in Tenm3/4-deficient synapses (Fig. 10f–i). Thus, the presynaptic Tenm3/4 deletion, different from the postsynaptic Tenm3/4 deletion, causes a major disruption of functional synaptic connectivity at MEC→CA1 synapses and additionally changed the properties of the remaining synapses.

## Discussion

Extensive studies have provided compelling evidence that teneurins are involved in the construction of synaptic circuits^1^. Mechanistically, the observation that teneurins clump cells when expressed in some types of tissue culture cells^29,32,51^ gave rise to the currently prevailing hypothesis that homophilic interactions between pre- and postsynaptic teneurins guide the specificity of synaptic connections^1,29^. However, two central questions regarding this hypothesis remained unanswered. First, are teneurins localized to synapses, or are they extrasynaptic adhesion molecules that primarily mediate other processes, such as neuronal migration and axon guidance? Second, is the function of teneurins in establishing synaptic connections required both pre- and postsynaptically, as predicted by the hypothesis of a homophilic mechanism of action, or do teneurins act as heterophilic adhesion molecules that are only essential in either pre- or postsynaptic neurons? The experiments we report here address both of these central questions.

In the first part of this project, we used confocal and STORM super-resolution microscopy to show that in the proximal CA1 region and the distal subiculum, Tenm3 (the teneurin isoform to which specific antibodies are available) is localized within the synaptic junctions (Figs. 1–3). We co-localized Tenm3 with both presynaptic (bassoon) and a postsynaptic marker (Homer1), and showed that it is equidistant to both (Figs. 2, 3). Strikingly, Tenm3 was not broadly present in synaptic junctions, but assembled into small intrasynaptic nanoclusters that exhibited a ~80 nm radius. Most synapses (>90%) contained at least one Tenm3 nanocluster, and half of the synapses included two or more Tenm3 nanoclusters (Figs. 2, 3). Since the synaptic junctions, as visualized both by Bassoon and Homer1 staining, had an average radius of 300 nm, the Tenm3 nanoclusters occupied less than 10% of the synaptic cleft. We and others previously showed that neurexins also assemble into similar nanoclusters in the synaptic cleft^47,52^, suggesting that these nanoclusters may be a general principle of synaptic adhesion molecules and complementing the nano-column organization of synapses^47,53,54^.

We observed that not all Tenm3 nanoclusters were associated with identifiable synaptic junctions. The non-synaptic Tenm3 nanoclusters we observed are unlikely to represent inhibitory synapses because these should also contain bassoon, and because we detected little co-localization of Tenm3 with the inhibitory synapse marker vGAT (Supplementary Fig. 1b, d), consistent with a low expression level of teneurins in inhibitory neurons (Supplementary Fig. 3). It is possible that the non-synaptic Tenm3 nanoclusters are associated with synaptic junctions that were not labeled because they are outside of the plane of focus. Alternatively, non-synaptic Tenm3 nanoclusters could be present in other neuronal or non-neuronal compartments (e.g., in astrocytes)^23,55^. In this respect, Tenm3 also resembles neurexins that exhibit a significant non-synaptic and non-neuronal component. Although teneurins are expressed in inhibitory neurons at lower levels and deletions of teneurins did not change the density of vGAT-positive inhibitory synaptic puncta in the hippocampal formation (Supplementary Figs. 6, 7), alternative splicing within the β-propeller domain can induce inhibitory synapses in heterologous synapse formation assays^6,7,35^. It is possible that teneurins have a functional impact on inhibitory synapses through interactions with specific postsynaptic binding partners that remain to be discovered, or that the heterologous synapse formation assay does not accurately reflect a physiological function of teneurins.

In the proximal CA1 region and distal subiculum, approximately half of the synaptic Tenm3 nanoclusters were ablated by deletion of Tenm3 from the MEC, proximal CA1 region, and distal subiculum, whereas a much lower proportion of non-synaptic Tenm3 nanoclusters were abolished, resulting in an increase in the relative prevalence of non-synaptic Tenm3 nanoclusters (Figs. 2, 3). The residual nanoclusters observed after the Tenm3 deletion in the MEC, proximal CA1 region, and distal subiculum may be contributed by synaptic projections from other brain regions. Viewed together, the super-resolution imaging analysis thus established that Tenm3 is an intrasynaptic protein that is organized in nanoclusters of excitatory synapses.

In the second part of this project, we asked whether teneurins perform an essential function at synapses, and whether their function is required in both pre- and postsynaptic neurons, or in only pre- or only postsynaptic neurons (Fig. 4). To address this question, we examined the effect on pre- or postsynaptic deletions of two teneurins*Tenm3* and *Tenm4*, on MEC→CA1 synapses, and in addition partly analyzed MEC→subiculum, MEC→dentate gyrus, and CA3→CA1 synapses. We opted to examine the double deletion of two teneurins (*Tenm3* and *Tenm4*) instead of single deletions of only *Tenm3* or *Tenm4* because of the potential for redundancy among teneurins. In our experiments, four different approaches, global synapse quantifications, SynaptoTag mapping, trans-synaptic pseudotyped rabies virus tracing, and electrophysiological recordings, established that presynaptic but not postsynaptic *Tenm3* and *Tenm4* are essential for various synapses formed by MEC neurons onto hippocampal targets (Figs. 5–8). Of these experiments, the retrograde pseudotyped rabies virus mapping approach and the electrophysiological recordings are probably the most definitive, as they reveal a completely normal synaptic connectivity after postsynaptic deletion of *Tenm3* and *Tenm4* in CA1 region neurons, but a severely impaired connectivity after presynaptic deletion of *Tenm3* and *Tenm4* in MEC neurons (Figs. 7–10).

Our data establish that Tenm3 and Tenm4 function as presynaptic adhesion molecules in hippocampal synapses, but are not required postsynaptically. These results are consistent with the finding that teneurins form high-affinity adhesion complexes with postsynaptic latrophilins^10–12,56^, and that postsynaptic (but not presynaptic) latrophilins are essential for synapse formation in CA1 region synapses^35,57^. A view thus emerges that teneurins organize synapses by a heterophilic interaction with latrophilins, but this view does not exclude additional functions for teneurins. Such additional function would also be consistent with the observation that teneurins have important roles in non-neuronal cells, as do latrophilins^15,19–24^. It is interesting that the presynaptic deletion of Tenm3/4 not only decreased the number of MEC→CA1 synapses, but also impaired the function of the residual synapses by suppressing their release probability (Fig. 10).

Finally, we would like to point out several limitations inherent in our approach. We only analyzed the localization of one teneurin isoform, Tenm3, in the hippocampus. It is possible that Tenm3 has a different localization in other brain regions, or that other teneurins have other localizations, although this seems unlikely given their structural and biochemical similarities. Moreover, we analyze the pre- vs. postsynaptic function of teneurins for only two isoforms (*Tenm3* and *Tenm4*), and only in a restricted number of synapses. It is thus conceivable, although again rather unlikely, that in other synapses teneurins function both pre- and postsynaptically. Since the experiments we report selectively blocked either only presynaptic or only postsynaptic teneurins, these experiments do not directly contradict previous data based on correlations of expression, but the conclusions are dramatically different^25,29,32^. Thus, although much remains to be done, our data are plausibly explained by a role for teneurins as heterophilic presynaptic adhesion molecules that contribute to synapse formation by binding to specific postsynaptic ligands, most likely latrophilins^35^. Moreover, we cannot exclude the possibility that the functions of *Tenm1* and *Tenm2*, which are co-expressed with *Tenm3* and *Tenm4* in the hippocampus and cortex (Supplementary Fig. 3), are redundant with those of *Tenm3* and *Tenm4* postsynaptically, but not presynaptically. If this were the case, pre- and postsynaptic teneurins would have to have the same functions postsynaptically but different functions presynaptically, which again seems implausible but cannot be ruled out. Finally, we did not reveal how teneurins function in synapse formation. Previous data showed that latrophilins act as GPCRs in synapse formation^58^, suggesting that teneurins may mediate synapse formation by controlling latrophilin GPCR activity, but no other evidence for this hypothesis is available. In addition, how teneurins transduce an intracellular signal that regulates the synapse formation remains to be explored. It is possible that the N-terminal intracellular domain (ICD) of teneurins translocates into the nucleus and acts as a transcriptional regulator^59,60^, or that teneurins organize the cytoskeleton.

In summary, we show that *Tenm3* is the synaptic protein that is assembled into nanoclusters in the synaptic cleft. These nanoclusters occupy a small part of a synaptic junction but are present in most synapses of its target area. Moreover, we show that *Tenm3* and *Tenm4* are jointly required presynaptically but not postsynaptically for the assembly of synaptic connections in the hippocampus. Thus, presynaptic teneurins likely interact with postsynaptic latrophilins and other ligands to organize synaptic connections, and via these interactions may contribute to establishing the specific wiring diagrams of neural circuits.

## Methods

### Mouse breeding, genotyping, and husbandry

All animal procedures conformed to the National Institutes of Health Guidelines for the Care and Use of Laboratory Animals and were approved by Administrative Panel on Laboratory Animal Care at Stanford University. *Tenm3* cKO mice were described previously^29^, and *Tenm4* cKO mice were a gift from Liqun Luo. Tenm3 and Tenm4 cKO mice were crossed to generate *Tenm3/4* DcKO mice. Mice were weaned at 20–21 days of age and group-housed (less than 5 mice per cage) on a 12 h light/dark cycle with food and water ad libidum, both male and female animals were used for all experiments.

The primer sequences used for genotyping were:

Tenm3 F: CTTGCTGGTTACTCTCGACA

Tenm3 R: ATAGCTGTGCACGTATGCAC

Tenm4 F: GACAACTGGCTACTCAACAGTAACATCC

Tenm4 R: GTGAGTCTATCAATTGGAACAACACATACC

### Plasmids and viruses

Two AAV-DJ vectors and two Lentivirus vectors were constructed. AAV viruses co-expressing NLS-∆Cre or NLS-Cre together with soluble tdTomato contained a P2A self-cleaving peptide driven by the human Synapsin promoter^61^. Lentivirus co-expressing NLS-HA-∆Cre or NLS-HA-Cre together with fusible tdTomato and Syb2-EGFP contained a P2A self-cleaving peptide and a T2A self-cleaving peptide under the EF1α promoter. The rabies complementing AAVs: AAV CAG FLEX TVA-mCherry and AAV CAG FLEX RG were gifts from Liqun Luo (Addgene #48332 and #48333).

### Lentivirus and AAV preparation

Lentiviruses and AAVs were prepared as described^49^. For productions of AAVs, briefly, AAV-DJ expressing vectors were co-transfected with the pHelper and pRC-DJ into HEK293T cells using calcium phosphate. Transfected cells were collected, lysed, and loaded into iodixanol gradient for ultracentrifugation (400,000 × *g* for 3 h). The 40% iodixanol fraction with the virus was further washed and concentrated with a 100,000 MWCO MWCO filter. The virus titer was measured by infecting HEK293 cells and stored in −80 °C before use.

For the production of Lentivirus, briefly, the lentiviral expressing vectors were co-transfected with the helper plasmids (pRSV-REV, pMDLg/pRRE, and vesicular stomatitis virus G protein (VSVG)) were cotransfected into HEK293T cell using calcium phosphate. After 48 h, cell media containing the lentiviruses were collected, filtered (0.45 µm pore size), and ultracentrifuged at 70,000 × *g* for 2 h. Pellets were resuspended in MEM and stored at −80 °C before use.

All viruses used in this paper are as below:

Lenti-Syn-∆Cre-EGFP^41^,

Lenti-Syn-Cre-EGFP^41^,

AAV-Syn-NLS-HA-∆Cre,

AAV-Syn- NLS-HA-Cre,

AAV-Syn-NLS-∆Cre-EGFP,

AAV-Syn- NLS-Cre-EGFP,

AAV-Syn-NLS-∆Cre-P2A-tdTomato,

AAV-Syn-NLS-Cre-P2A-tdTomato

AAV-CAG-Flex-RG,

AAV-CAG-Flex-TCB,

AAV-Syn-TCB-mCherry-P2A-RG,

RbV-CVS-N2c-deltaG-GFP (EnvA),

Lenti-EF1a-NLS-HA-∆Cre-P2A-tdTomato-T2A-EGFP::Syb2,

Lenti-EF1a-NLS-HA-Cre-P2A-tdTomato-T2A-EGFP::Syb2.

### Slice electrophysiology

For acute slice electrophysiology, AAVs were unilaterally injected in the MEC or proximal CA1 region into P0 pups, and CA1 pyramidal neurons were recorded at P19-22. Control (∆Cre-injection) and test (Cre-injection) groups were conducted in a blind manner within the same day. The brains were removed and transverse hippocampal sections (250 µm) were sliced with a vibratome in ice-cold dissection buffer containing the following (in mM): 228 Sucrose, 26 NaHCO_3_, 11 D-Glucose, 2.5 KCl, 1 NaH_2_PO_4_, 7 MgSO_4_ and 0.5 CaCl_2_ saturated with 95% O_2_ and 5% CO_2_. Sections were allowed to recover in artificial cerebrospinal fluid (ACSF) containing (in mM): 119 NaCl, 26 NaHCO_3_, 11 D-Glucose, 2.5 KCl, 1 NaH_2_PO_4_, 1.3 MgSO_4_, and 2.5 CaCl_2_ (continuously equilibrated with 95% O_2_ and 5% CO_2_) at 30 °C for 30 min, followed by holding at room temperature for 1 h. Whole-cell recordings were made using an internal solution containing (in mM): 140 CsMeSO_4_, 8 CsCl, 10 HEPES, 0.25 EGTA, 2 MgATP, 0.3 Na3GTP, 0.1 spermine, 7 phosphocreatine (pH 7.25–7.3; osmolarity 294–298). AMPA-EPSCs were evoked by electrical stimulation using a bipolar electrode (FHC, USA) positioned at the stratum radiatum proximal for CA3→CA1 Schaffer collateral recording, and the stratum lacunosum-moleculare proximal to the dentate gyrus for MEC→CA1 perforant path recording. Picrotoxin was included in extracellular ACSF in all experiments examining excitatory synaptic transmission. Stimulation pulses were delivered every 15 s. AMPAR EPSCs were recorded with a holding potential of −70 mV, whereas NMDAR EPSCs were recorded at +40 mV and quantified at 50 ms after the stimulus artifact. Two pulses with a 50 ms inter-stimulus interval were delivered to calculate paired-pulse ratios (PPRs). All drugs were obtained from Tocris (Minneapolis, MN, USA).

### Junction-flanking PCR

To determine the mRNA levels of Teneruins in different brain regions (CA1 region and MEC region) and ages (P12 and P21), brain regions of interest were microdissected from the fresh wildtype mice brains. RNA extraction was performed using the QIAGEN RNeasy kit. Alternative splicing of Teneruins mRNA was analyzed using junction-flanking PCR. The following primers anneal to constitutive exon sequences that flank splice junctions and thus amplify Teneurins mRNA transcripts with or without alternative splice sequences (gene, forward primer, reverse primer): Tenm3 (5′ -ATTACGTCCGGCGGATATT-3′, 5′-GTAGCCAGGTAGTATCTGTGAG-3′), Tenm4 (5′-GACTTCAACTACATCCGCAGAA-3′, 5′-GTTGGTGTCAGACAGGAAGAC-3′). Junction-flanking PCR was performed on cDNA synthesized from equal amounts of RNA using the SuperScript™ IV First-Strand Synthesis System (Invitrogen Cat# 18091050). Running conditions were optimized to ideal resolution and band separation for quantification, due to the different molecular weights of the PCR products. Samples were loaded on 2% MetaPhorTM Agarose gels were stained using PAGE GelRed^®^ Nucleic Acid Gel Stain (Biotium Cat# 41008). Stained gels were imaged at subsaturation using the ChemiDoc Gel Imaging System (Bio-Rad). Quantification was analyzed by ImageJ. Intensity values were normalized to the size of DNA products to negate intensity differences related to increased dye incorporation with increased DNA length.

### Monosynaptic retrograde rabies tracing and stereotactic injections

For synaptic marker and SynaptoTag stereotactic injection experiments, P0 pups were anesthetized on ice for 3–5 min and fixed on an ice bag with ear bars. Injection coordinates were zeroed from lambda. 0.15 µl of 10^8^ IU/mL titer AAV virus or 0.4 µl of 10^8^ IU/mL lentivirus were injected in MEC region, 0.06 µl of 10^8^ IU/mL titer AAV virus were injected at proximal CA1 using a glass micropipette connected a 10 µl Hamilton syringe completely continuous with mineral oil. The coordinates used for the MEC were AP −0.45 mm, ML +/−2.05 mm, DV −1.85 mm. The coordinates used for proximal CA1 (p-CA1) were AP +0.55 mm, ML +/−2.05 mm, DV −1.85 mm. The coordinates used for the distal subiculum (d-Sub) were AP +0.15 mm, ML +/−1.80 mm, DV −1.85 mm. Pups were allowed to recover on a heating pad in the new cage after injection and were transferred back to their home cage after recovering.

For the retrograde trans-synaptic rabies tracing experiments^50^, 0.2 µl of lentivirus expressing NLS-eGFP-Cre were injected unilaterally at proximal CA1 in P0. Complementing AAVs containing CAG-FLEX-TCB-mCherry and CAG-FLEX-RG were generated from the Janelia Farm Viral Core Facility, and 0.4 µl of a 1:1 mixture of them were injected at proximal CA1 in P21 mice. The coordinates were zeroed from bregma and used for proximal CA1 were AP −2.7 mm, ML +/−3.8 mm, DV −2.9 mm. Adult mice were anesthetized by injection of Avertin (250 mg/kg) and head-fixed using stereotaxic equipment. After two weeks, 0.4 µl glycoprotein-deleted rabies virus (EnvA) was produced generated from the Janelia Farm Viral Core Facility, and injected into the same brain region at proximal CA1 in P35 mice. One week later, mice were perfused and analyzed for imaging in P41. After injection, all adult mice were allowed to completely recover on a heating pad in a clean cage.

For presynaptic rabies tracing approach, 0.15 µl of 10^8^ IU/mL titer AAVs expressing NLS-HA-Cre or NLS-HA-∆Cre were injected unilaterally at MEC neurons in P0. The single AAVs helper vector AAV-TCB-mCherry-P2A-RG was constructed, 0.06 µl viruses were injected at proximal CA1 in P21 mice. Adult mice were anesthetized by injection of Avertin (250 mg/kg) and head-fixed using stereotaxic equipment. After two weeks, 0.2 µl glycoprotein-deleted rabies virus (EnvA) was produced generated from the Janelia Farm Viral Core Facility, and injected into the same brain region at proximal CA1 in P35 mice. One week later, mice were perfused and analyzed for imaging in P41. After injection, all adult mice were allowed to completely recover on a heating pad in a clean cage.

### Immunohistochemistry

All solutions were made fresh and filtered via a 0.22 µm filter prior to starting experiments. Mice were briefly anesthetized by isoflurane and perfused transcardially with PBS followed by ice cold 4% paraformaldehyde (by weight) in PBS. Brains were dissected, post-fixed in 4% paraformaldehyde for either 1 h (for Tenm3 and synaptic marker staining) or overnight at 4 °C for tracing staining or no staining, washed with PBS for three times, cryoprotected for 48 h in 30% sucrose at 4 °C. The brains were then embedded in Optimum Cutting Temperature (OCT, Tissue Tek) and stored at −80 °C until being sectioned. Horizontal sections (40 µm) were collected at −20 °C using a cryostat. The free-floating sections were collected and washed with PBS and blocked for 1 h in blocking buffer (4% BSA/3% normal goat serum/0.3% Triton-X100/0.05% sodium azide/PBS) at room temperature. The primary antibodies (Guinea pig anti-vGluT11, Millipore Cat# AB5905, Rabbit anti-vGluT1, TCS Cat# YZ6089, Guinea pig anti-vGAT Millipore Cat# AB5062P, Guinea pig anti-vGATSynaptic Systems Cat# 131004, Mouse anti-Bassoon Abcam Cat# 82958, Mouse anti-Homer1 Synaptic SystemsCat# 160011, Rabbit anti-Homer1Synaptic SystemsCat# 160003, Chicken anti-GFPAves Labs Cat# 1020, Chicken anti-GFP Invitrogen Cat# A10262, Rat anti-mCherry, Invitrogen Cat# M11217, Rabbit anti-HA Cell Signaling Technologies Cat# 3724, Rabbit anti-Teneurin3 (IC)^29^)were diluted in blocking solution and brain sections were incubated with primary antibody for 24–96 h at 4 °C, followed by three 15 min washes in PBS and 2 h incubation with secondary antibodies (Goat anti-Chicken IgY (H + L) Secondary Antibody, Alexa Fluor 488, Invitrogen Cat# A11039, Goat anti-Guinea Pig IgG (H + L) Highly Cross-Adsorbed Secondary Antibody, Alexa Fluor 647, Invitrogen Cat# A-21450, Goat anti-Rat IgG (H + L) Cross-Adsorbed Secondary Antibody, Alexa Fluor 546, Invitrogen Cat# A-11081, Goat anti-Rabbit IgG (H + L) Highly Cross-Adsorbed Secondary Antibody, Alexa Fluor 647, Invitrogen Cat# A-21245, Highly cross-adsorbed donkey anti-mouse IgG (H + L) secondary antibody CF568, Biotium, 20105) diluted in blocking buffer at room temperature. For Tenm3 staining, brain sections underwent antigen retrieval in 10 mM sodium citrate for 30 min before incubation with primary antibodies. Sections were labeled with DAPI (Millipore, Cat# 10236276001) diluted into PBS for 20 min at room temperature, washed 15 min for three times with PBS, and mounted on glass slides coated in 0.1% Triton-X100/PBS, dried, and coverslipped with Fluoromount-G (Southern Biotech #010001).

### Confocal image acquisition and analysis

Serial confocal z-stack images were acquired using a Nikon confocal microscope (A1RSi) with a 10×, 20×, 60×, and 100× objective. Images were analyzed using NIS-Elements AR acquisition software. All staining processes and acquisition parameters were kept constant among different experiments. For synaptic puncta quantification, z-stack images were acquired at 0.2 µm intervals and five consecutive sections with the highest signal were projected maximally. Six regions-of-interest were imaged and analyzed in different experiments conditions: proximal CA1 (p-CA1), distal CA1 (d-CA1), proximal subiculum (p-sub), distal subiculum (d-sub), dentate gyrus (DG), and MEC. The background subtraction was measured within each set of the experiments, and the same background threshold was applied to each set of experiments and puncta parameters were automatically obtained using the analysis software Nikon NIS-Elements. Dotted boxes indicate areas that were quantitatively analyzed in Fig. 5a–c, g–i, Supplementary Figs. 4–7. The S. lacunosum-moleculare of the proximal CA1 (p-CA1) and the molecular layer of distal subiculum (d-sub) were used as experimental regions, the S. lacunosum-moleculare of the proximal CA1 (p-CA1) and the molecular layer of distal subiculum (d-sub) were used as inner control regions. For rabies tracing quantification as described^35^, briefly, horizontal sections (60 µm) were labeled with DAPI and imaged using a 10× and 20× objective, four regions-of-interest were collected: the proximal CA1 (starter cells), ipsilateral CA3, ipsilateral MEC, and contralateral CA3. Cells were countered using Nikon NIS-Elements AR acquisition software and ImageJ in each region-of-interest.

### Direct stochastic optical reconstruction microscopy (dSTORM) imaging

Direct Stochastic Optical Reconstruction Microscopy (dSTORM) images were acquired using a Vutara SR 352 (Bruker Nanosurfaces, Inc., Madison, WI) commercial microscope based on single molecule localization biplane technology^62–64^. A light fixation protocol was used to improve the specificity of labeling for dSTORM. P13 mice were briefly anesthetized by isoflurane and perfused transcardially with PBS (5 ml) followed by ice cold 4% paraformaldehyde in PBS (10 ml). Brains were dissected, and the interested hippocampal regions were collected and post-fixed in 4% paraformaldehyde for 15–20 min, washed with PBS for three times, cryoprotected for 48 h in 30% sucrose at 4 °C until being sectioned. 25 µm thick hippocampal sections were collected as described and labeled with mouse anti-Bassoon (Abcam # 82958;1:250), mouse anti-Homer1 (Synaptic Systems # 160011; 1:250), rabbit anti-Teneurin3 IC^29^ (1:1,000) primary antibodies and secondary antibodies conjugated to Alexa647 (Thermo Fisher 1:2000) or CF568 (Biotium 1:2,000). All solutions were made fresh and filtered via a 0.2 µm filter prior to starting experiments and wash buffers included 0.03% Triton-X100. The brain sections were incubated with 100 mM glycine in PBS for 10 min before staining. The labeled sections were mounted on a coverslip treated with poly-L-Lysine and placed in dSTORM buffer containing (in mM) 50 Tris-HCl at pH 8.0, 10 NaCl, 20 MEA, 1% β-mercaptoethanol, 10% glucose, 150 AU glucose oxidase type VII (Sigma # G2133), and 1500 AU catalase (Sigma #C40). Labeled proteins were imaged with 647 nm and 561 nm excitation power of 40 kW/cm^2^. Images were recorded using a 60×/1.2 NA Olympus water immersion objective and Hamamatsu Flash4 sCMOS camera with gain set at 50 and frame rate at 50 Hz. Data were analyzed by Vutara SRX software (version 6.04). Single molecules were identified in each frame by their brightness after removing the background. Teneurin3 was imaged for 10,000 frames with the first 3000 frames excluded from analysis. Bassoon was imaged for 7000 frames with the first 2000 frames excluded from analysis. Homer1 was imaged for 7000 frames with the first 1000 frames excluded from analysis. Identified molecules were localized in three dimensions by fitting the raw data in a 12 × 12-pixel region of interest centered around each particle in each plane with a 3D model function that was obtained from recorded datasets of fluorescent beads. Fit results were filtered by a density based denoising algorithm to remove isolated particles and rendered as 50 nm points. The remaining localizations were classified into clusters by density-based spatial clustering of applications with noise (DBSCAN), a minimum of 10 localizations were connected around a 100 nm search radius for Teneurin-3 and 50 localizations around a 300 nm search radius for Bassoon and Homer1. A standard 100 nm hull was used for both. Data were filtered using STORM RLA (settings: 20 px/μm, no limit, 200 Monte Carlo, 500 nm radius, 100 bins). The experimentally achieved image resolution of 40 nm laterally (*xy*) and 70 nm axially (*z*) was determined by Fourier ring correlation. For analysis the distribution of Tenm3 nanoclusters and Bassoon or Homer1 localizations in Supplementary Fig. 2B, E, histograms were fitted with the following functions in Matlab: Gaussian: *f*(*x*) = *a*1*exp(−((*x* − *b*1)/*c*1)^2^) + *a*2*exp(−((*x* − b2)/*c*2)^2^); other images were processed using ImageJ and Matlab.

### Data analyses

All data are shown as means ± SEM, numbers of sections and mice analyzed are shown in the bars or plots. Statistical significance (*p < 0.05, **p < 0.01, ***p < 0.001, non significant comparisons are not indicated) was analyzed with Prism 8, GraphPad. Two-tailed Student’s t-test was used for comparison between the two groups. One-way ANOVA was used for comparison among more than two groups. Two-way ANOVA was used for the comparison of multiple groups with multiple factors. All experiments were performed and analyzed in a blind manner by the experimenter.

### Reporting summary

Further information on research design is available in the Nature Research Reporting Summary linked to this article.

## Supplementary information

Supplementary Information

Reporting Summary

## Data Availability

All relevant data supporting the findings of this study are available from the corresponding authors upon request. Source data are provided with this paper. Heat maps in Supplementary Fig. 3 of NAUC (normalized area under the curve) expression values were obtained from the ASCOT (Alternative Splicing & Gene Expression Summaries of Public RNA-Seq Data) database (http://ascot.cs.jhu.edu/). Source data are provided with this paper.

## Source data

Source Data

## Code availability

All relevant code supporting the findings of this study is available from the corresponding authors upon request.
