# Author Correction: Teneurins assemble into presynaptic nanoclusters that promote synapse formation via postsynaptic non-teneurin ligands

**DOI:** 10.1038/s41467-023-38713-0

**Published:** 2023-05-23

**Authors:** Xuchen Zhang, Pei-Yi Lin, Kif Liakath-Ali, Thomas C. Südhof

**Affiliations:** 1 Howard Hughes Medical Institute, Stanford University, Stanford, CA USA; 2 Department of Molecular and Cellular Physiology, Stanford University, Stanford, CA USA

**Keywords:** Molecular neuroscience, Neural circuits

Correction to: *Nature Communications* 10.1038/s41467-022-29751-1, published online 28 April 2022

The original version of the Source Data file associated with this Article included errors in the data shown for figure 8b and figure 8k.

The Source Data for fig. 8b incorrectly contained an additional column of data, under ‘AB’, which was incorrectly pasted from elsewhere. This column has been removed from the Source Data for Fig. 8b.

The values shown in the Source Data for Fig. 8k column AC were incorrect, as these were inadvertently pasted from Fig. 8k column M. The Source Data for Fig. 8k, column M has been corrected.

The HTML has been updated to include a corrected version of the Source Data file; the original incorrect version of the Source Data file can be found as Supplementary Information associated with this Correction.

## Supplementary information

Updated Source Data

Incorrect Source Data

