# Supplementary material for: Teneurins assemble into presynaptic nanoclusters that promote synapse formation via postsynaptic non-teneurin ligands
Source: Nat Commun. 2022 Apr 28;13:2297. doi: 10.1038/s41467-022-29751-1 (PMC9050732; doi:10.1038/s41467-022-29751-1)
Supplement: Supplementary file 1 — Supplementary Information [file 41467_2022_29751_MOESM1_ESM.pdf]

Supplementary Figures

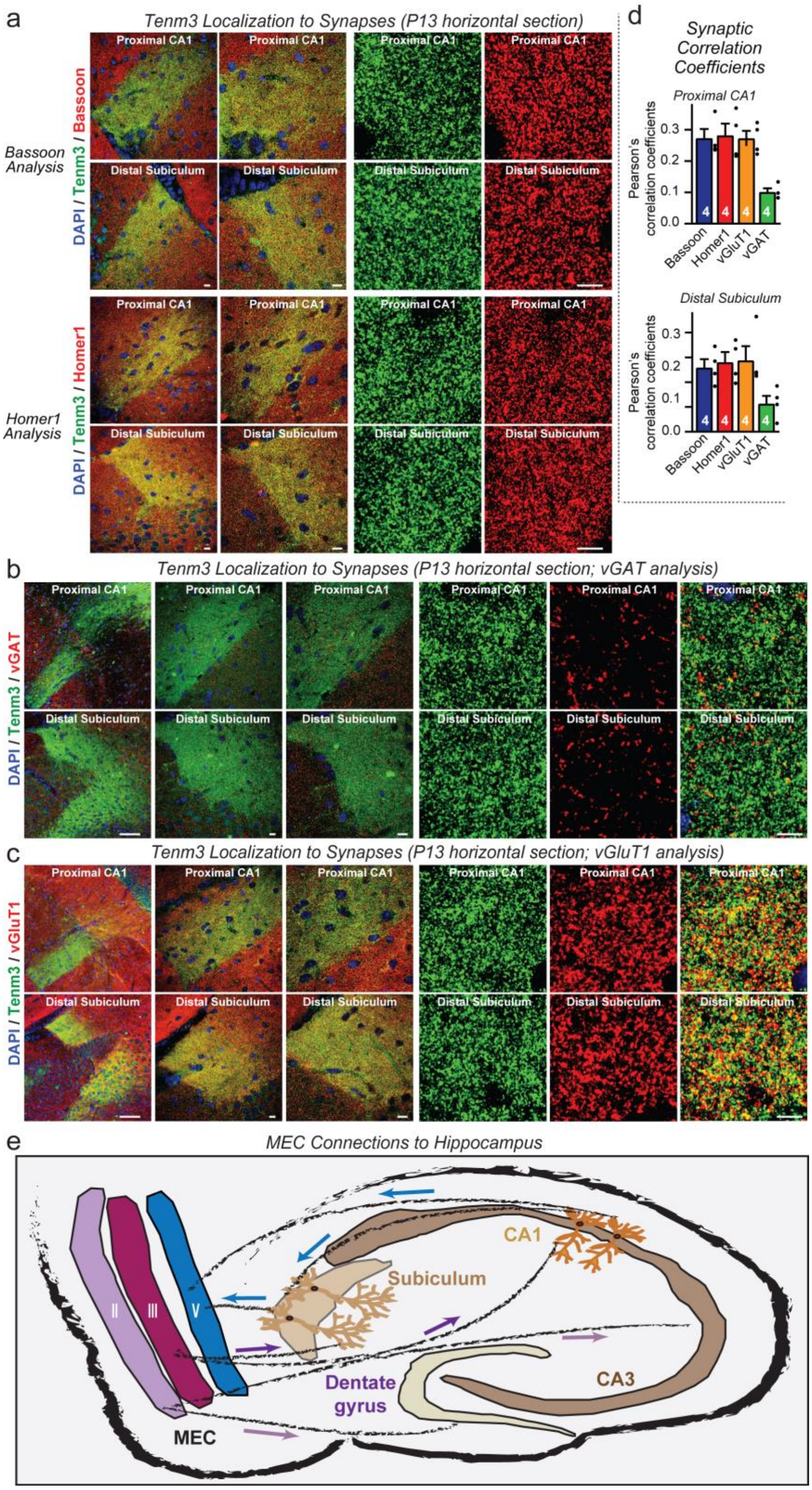

Supplementary figure 1: Teneurin-3 (Tenm3) is a synaptic protein (related to Fig. 1). a Low- and high-resolution images show that Tenm3 puncta overlap with those of the presynaptic active zone marker, Bassoon (top two rows) and of the postsynaptic scaffolding protein, Homer1 (bottom two rows) in the S. lacunosum-moleculare of the proximal CA1 (top row in each group) and the molecular layer of distal subiculum (bottom row in each group). Data complement those shown in Fig. 1b. **b** Low- and high-resolution images show that Tenm3 puncta in the S. lacunosum-moleculare of the proximal CA1 (top) and in the

molecular layer of distal subiculum (bottom) do not overlap with vGAT-positive puncta that correspond to inhibitory synapses. **c** Low- and high-resolution images show that Tenm3 puncta in the S. lacunosum-moleculare of the proximal CA1 (top) and the molecular layer of distal subiculum (bottom) overlap with vGluT1-positive puncta marking excitatory synapses. **d** Summary graphs of the correlation coefficients of the Tenm3 signal with those of the indicated synaptic markers (Bassoon, Homer1, vGluT1, and vGAT) in the S. lacunosum-moleculare of the proximal CA1 (top) and the molecular layer of distal subiculum (bottom) show that the Tenm3 signal is associated with excitatory but not inhibitory synapses. **e** Schematic of the topographic synaptic connections linking the MEC, proximal CA1, distal subiculum, dentate gyrus and CA3 to each other. MEC neurons project to proximal CA1, distal subiculum, and dentate gyrus/CA3, proximal CA1 neurons project to the distal subiculum and MEC, and distal subiculum neurons send axons to the MEC. Note that in the horizontal sections used for the analyses in the current study, not all of the projections will be visualized. Data are means ± SEM; numbers of sections/mice are indicated in the bars. Scale bars: 10 µm (**a**, left two panels), 5 µm (**a**, right two panels), 100 µm (**b**, **c**, left three panels), 5 µm (**b**, **c**, right three panels).

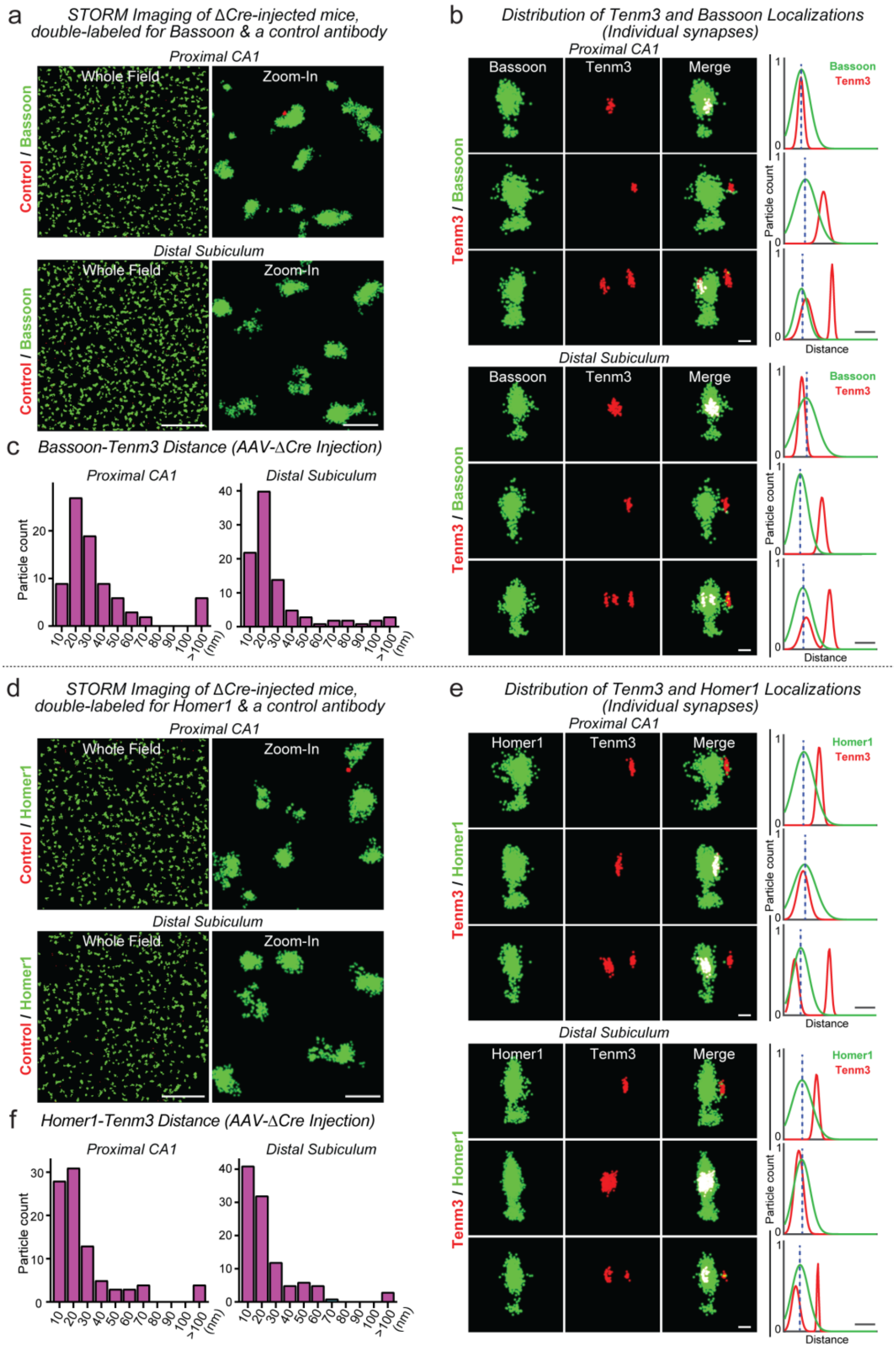

Supplementary figure 2: Super-resolution STORM imaging of hippocampal sections double-labeled for Tenm3 and Bassoon or Homer1 identifies synaptic Tenm3 nanoclusters (related to Fig. 2 and 3). a Labeling of sections with a control antibody instead of Tenm3 antibodies. Representative STORM images show that are control antibody (red) does not produce nanocluster signals generated by staining with Tenm3 antibodies (green, Bassoon-positive macroclusters corresponding to presynaptic active zones; left, overview; right, high-resolution images). Data are from WT mice at P13. **b** Illustration of the relative positions of Tenm3 nanoclusters and Homer1 macroclusters as visualized by high-

resolution STORM images. Representative images are shown on the left (red, Tenm3 nanoclusters; green, Bassoon macroclusters), and the distribution of the Tenm3 and Bassoon signal along the distance from the center of mass in a 3D space is graphically depicted on the right for each representative image (top rows, proximal CA1 region; bottom rows, distal subiculum). Examples were chosen to illustrate different relationships between Tenm3 nanoclusters and Bassoon-positive presynaptic active zones. **c** Histogram of the Bassoon-Tenm3 distances in individual synapses in the proximal CA1 (left) and distal subiculum (right). Note that most of the Tenm3 nanoclusters are less than 30 nm away from the Bassoon macroclusters in both the proximal CA1 and distal subiculum. **d-f** Same as **a-c**, except that sections were analyzed by double-labeling with Tenm3 (red) and Homer1 antibodies (green). Again, note that most of the Tenm3 nanoclusters are less than 30 nm away from the Homer1 macroclusters in both the proximal CA1 and distal subiculum. Scale bars: 10 µm (**a**, **d**, left panel), 1 µm (**a**, **d**, right panel), 200 nm (**b**, **e**).

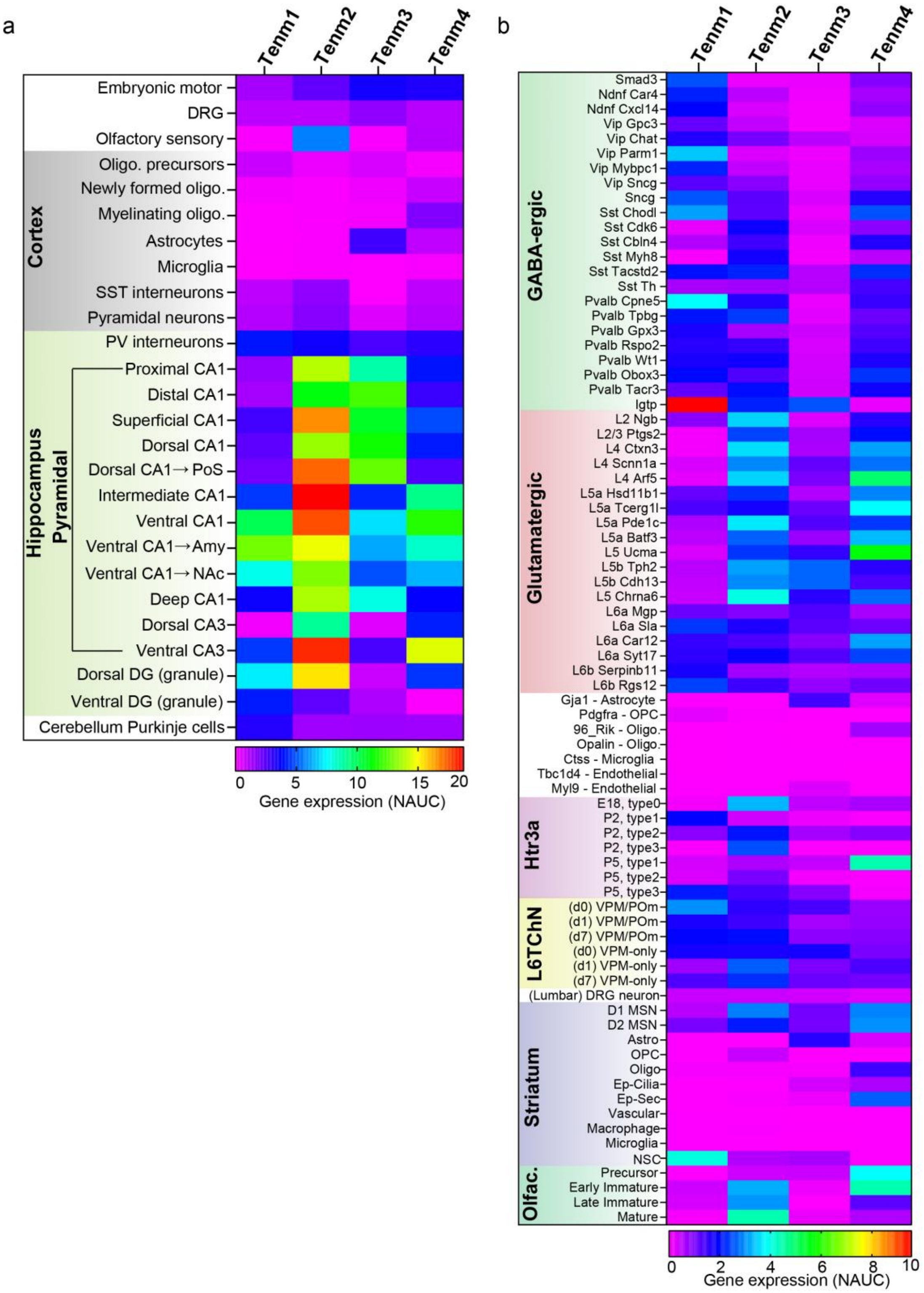

Supplementary figure 3: Multiple teneurins are co-expressed in excitatory neurons in the forebrain as revealed by published RNA-seq data. a Heat map showing expression of teneurins from bulk RNA-seq datasets. **b** Heat map showing expression of teneurins from single-cell RNA-seq datasets in cortical, striatum and olfactory bulb neurons. NAUC (normalized area under the curve) expression values were obtained from the ASCOT (Alternative Splicing & Gene Expression Summaries of Public RNA-Seq Data) database (http://ascot.cs.jhu.edu/) (Ling et al., Nat Comm 2020).

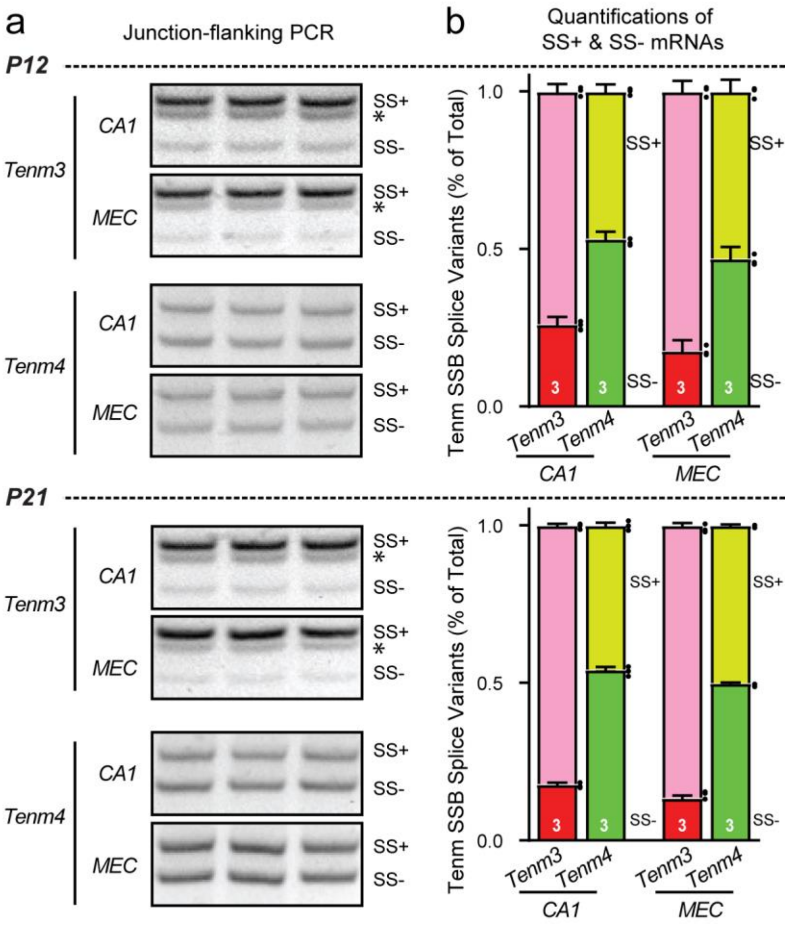

Supplementary figure 4: Tenm3 and Tenm4 are expressed in hippocampal formation with the SSB- splice variant that binds to latrophilins. a, Representative gel images show Tenm3 and Tem4 alternative splicing at SSB in CA1neurons and MEC regions at P12 (top rows) and P21 (bottom rows). Each panel represents a single site of alternative splicing (*, non-specific band). **b**, Alternative splicing was quantified using junction-flanking PCR, as distribution of splice variants in % of the total. Bar graphs show means ± SEM; numbers of mice are indicated in the bars.

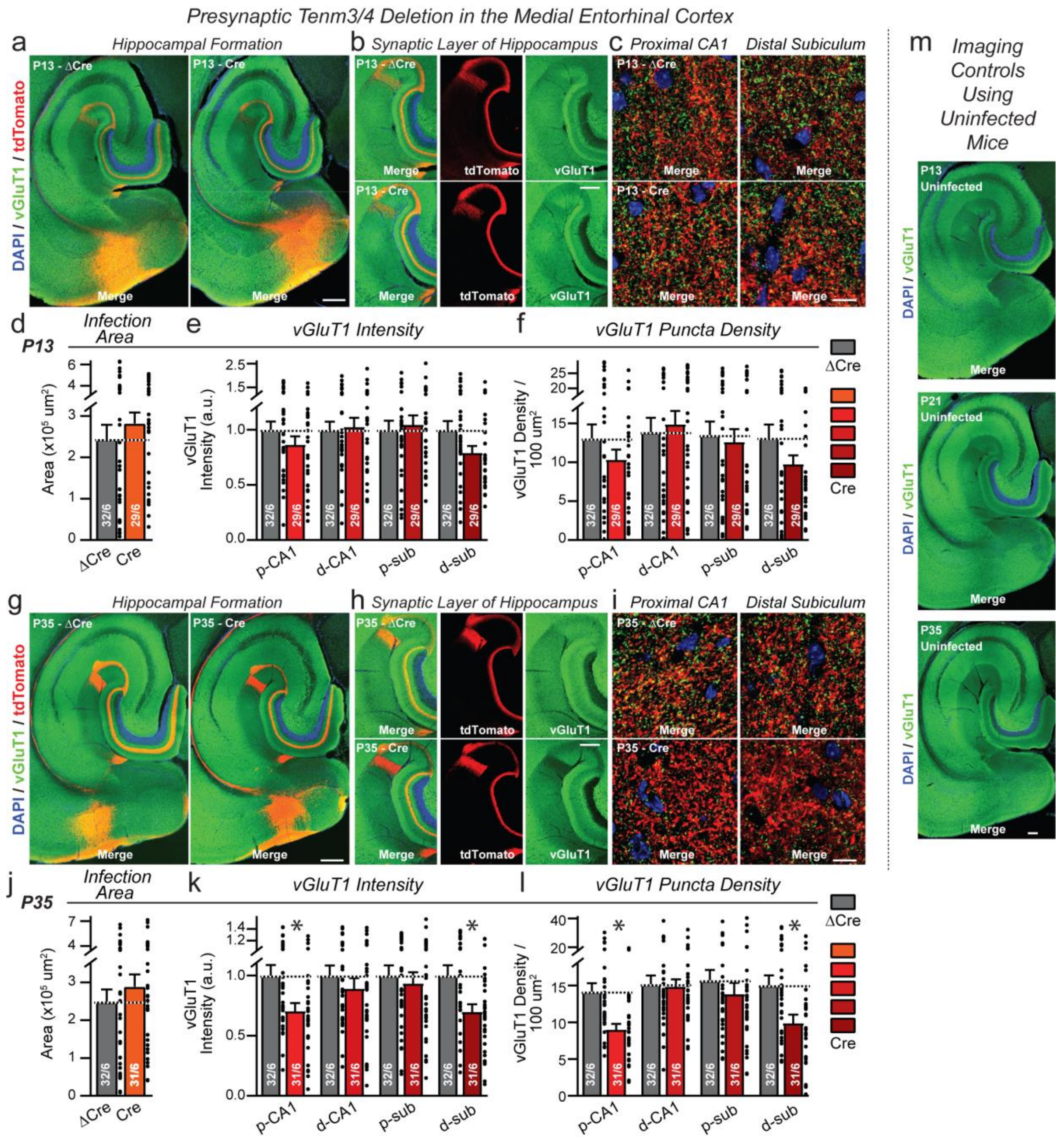

Supplementary figure 5: Analysis of the effect of presynaptic Tenm3/4 deletions in the MEC on the synapse density in the CA1 and subiculum at P13 and at P35 (related to Fig. 4). All data were from mice whose MEC was infected at P0 with AAVs expressing ΔCre (control) or Cre. All sections were stained for vGluT1 as a presynaptic marker of excitatory synapses. **a** Representative overview images of hippocampal sections at P13. **b** Representative low-resolution images of vGluT1 stained hippocampal sections at P13. **c** Representative high-resolution images of vGluT1 staining in the S. moleculare-lacunosum of the proximal CA1 (left) and the molecular layer of distal subiculum (right) at P13. **d** Quantifications showing that the MEC areas infected with ΔCre- and Cre-expressing AAVs, as measured via the tdTomato signal, were not significantly different at P13. **e,f** Presynaptic deletion of Tenm3/4 in MEC neurons causes a modest non-significant decrease in the overall density of excitatory synapses in the areas to which the MEC neurons project as revealed by their tdTomato signal, namely the proximal CA1 (p-CA1) and distal subiculum (d-sub). The distal CA1 (d-CA1) and proximal subiculum (p-sub), which are hippocampal areas lacking tdTomato-positive MEC projections, exhibit no change in synapse density.

Summary graphs depict quantifications of the global vGluT1 staining intensity (**e**) and the vGlut1-positive puncta density (**f**) as two measures of synapse density. **g-l** Same as **a-f**, except that brain regions of proximal CA1 and distal subiculum were analyzed at P35, and that the synapse density decrease becomes significant in the areas receiving synaptic projections from the MEC. **m** Imaging controls using uninfected mice. Bar graphs show means ± SEM; numbers of sections/mice are indicated in the bars. Statistical significance was assessed using two-sided Student's t-test and Two-way ANOVA (*, $p<0.05$). Scale bars: 1 mm (**a**, **g**), 0.2 mm (**b**, **h**), 10 µm (**c**, **i**) and 0.5 mm (**m**).

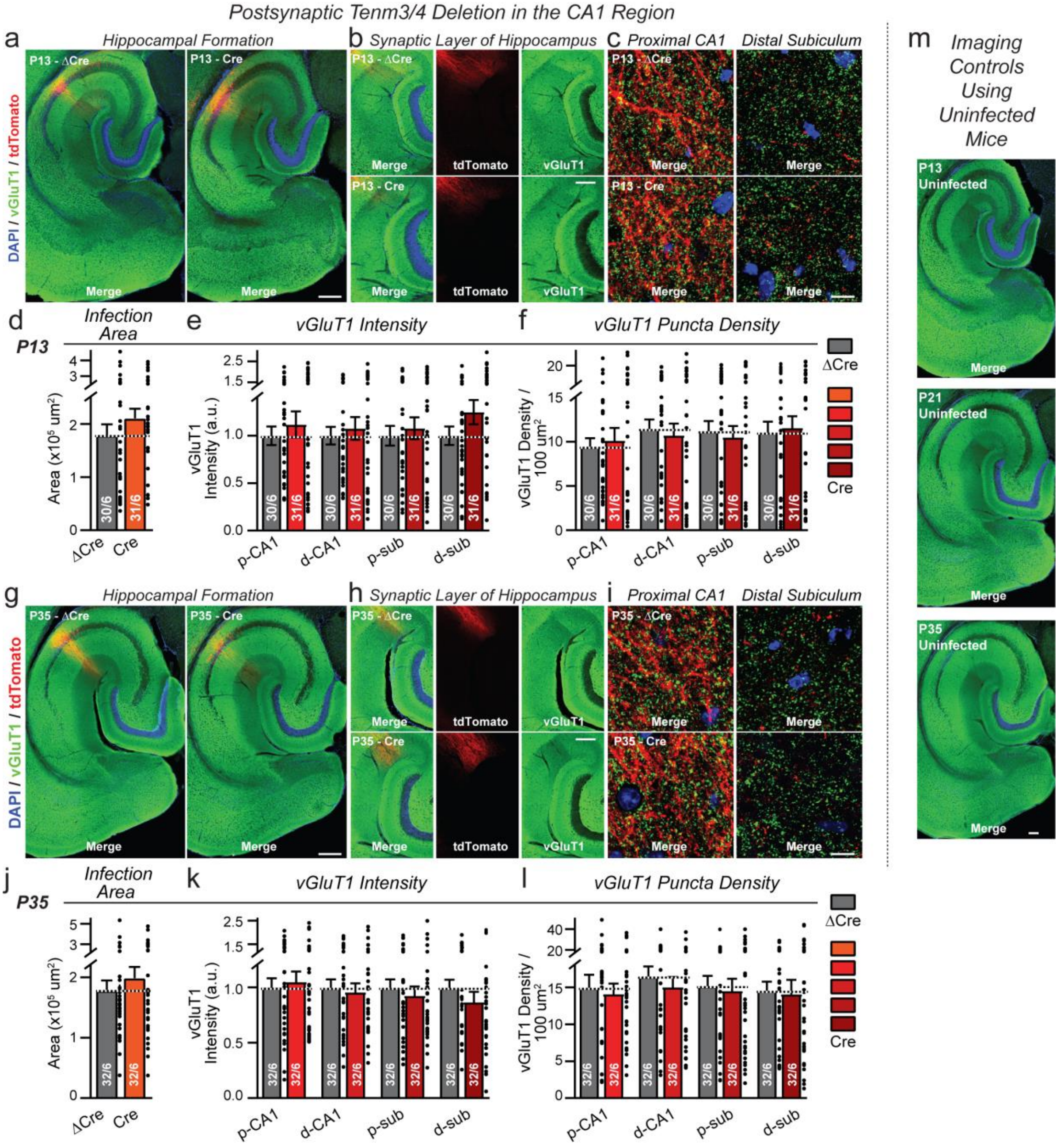

Supplementary figure 6: Analysis of postsynaptic Tenm3/4 deletions in the proximal CA1 neurons– effect on synapse density in the CA1 and subiculum at P13 and P35 (related to Fig. 4). All data were from mice whose CA1 neurons were infected at P0 with AAVs expressing ΔCre (control) or Cre. All sections were stained for vGluT1 as a presynaptic marker of excitatory synapses. **a** Representative overview images of hippocampal sections at P13. **b** Representative low-resolution images of vGluT1-stained hippocampal sections at P13. **c** Representative high-resolution images of vGluT1 staining in the S. moleculare-lacunosum of the proximal CA1 (left) and the molecular layer of distal subiculum (right) at P13. **d** Quantifications showing that the CA1 areas infected with ΔCre- and Cre-expressing AAVs, as measured via the tdTomato signal, were not significantly different at P13. **e, f** Postsynaptic deletion of Tenm3/4 in MEC neurons has no effect on the overall density of excitatory synapses at P13 in the areas to which the MEC neurons project. Summary graphs depict quantifications of the global vGluT1 staining intensity (**e**) and the vGlut1-positive puncta density (**f**) as two measures of synapse density. **g-l** Same as **a-f**, except that brain regions of proximal CA1 and distal subiculum were analyzed at P35. **m**

Imaging controls using uninfected mice. Bar graphs show means ± SEM; numbers of sections/mice are indicated in the bars. Statistical significance was assessed using two-sided Student's t-test and Two-way ANOVA (non-significant comparisons are not indicated). Scale bars: 1 mm (**a**, **g**), 0.2 mm (**b**, **h**), 10 µm (**c**, **i**) and 0.5 mm (**m**).

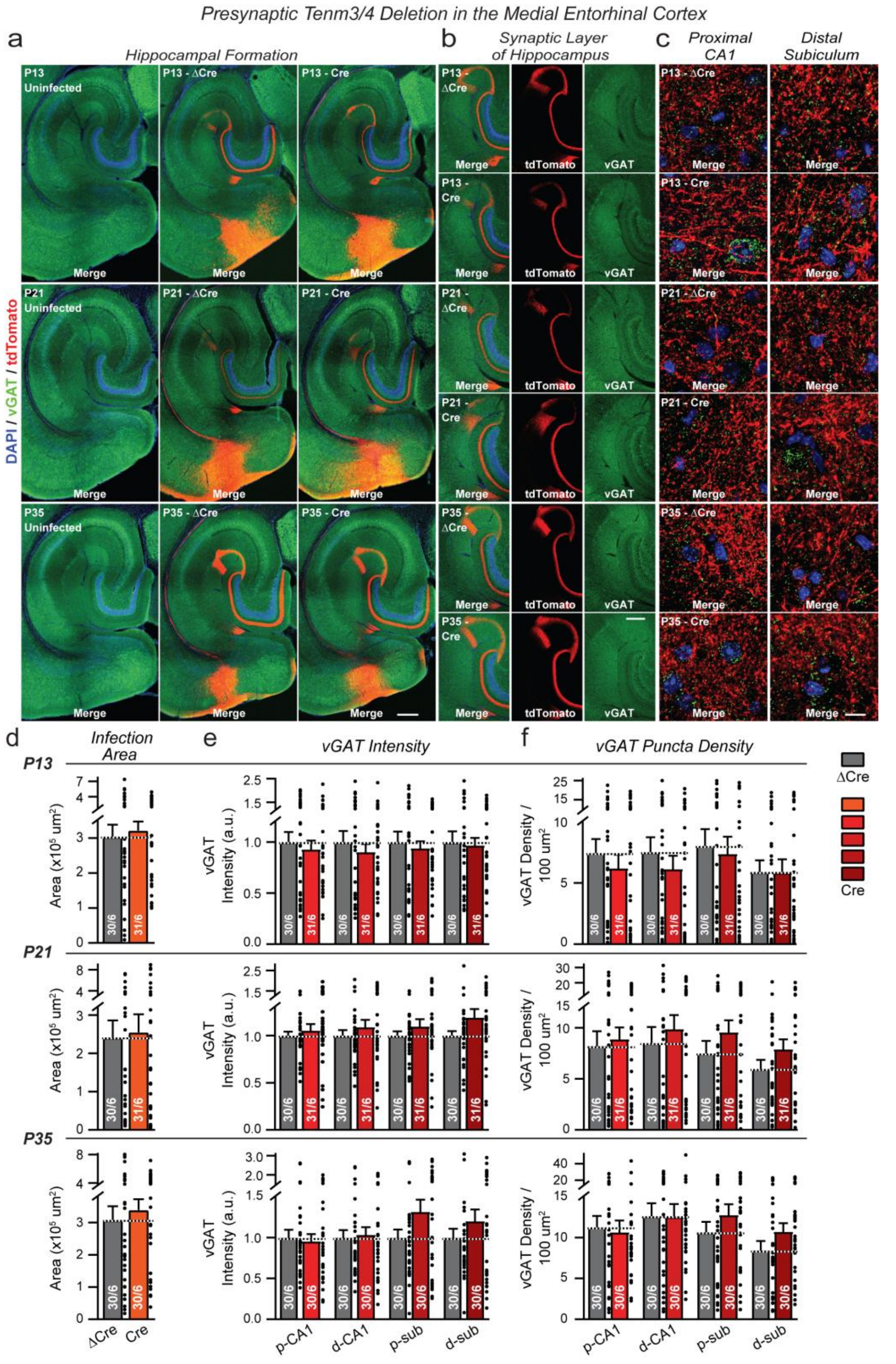

Supplementary figure 7: Deletion of Tenm3 and Tenm4 in the MEC neurons has no effect on inhibitory synapse density in the CA1 and subiculum (related to Fig. 4). All data were from mice whose MEC neurons were infected at P0 with AAVs expressing ΔCre (control) or Cre. All sections were stained for vGAT as a presynaptic marker of inhibitory synapses. **a** Representative overview images of hippocampal sections from uninfected mice (left) and mice infected with AAVs expressing ΔCre (center) or Cre (right), examined at P13 (top row), P21 (middle row), or P35 (bottom row). **b** Representative low-resolution images of vGAT stained hippocampal sections corresponding to the overviews shown in **a**. **c**

Representative high-resolution images of vGAT staining in the S. moleculare-lacunosum of the proximal CA1 (left) and the molecular layer of distal subiculum (right) at P13, P21, and P35. **d** Quantifications showing that the MEC areas infected with ∆Cre- and Cre-expressing AAVs, as measured via the tdTomato signal, were not significantly different at P13, P21, and P35. **e, f** Presynaptic deletion of Tenm3/4 in MEC neurons causes no change in the overall density of inhibitory vGAT-positive synapses in the areas to which the MEC neurons project. Summary graphs depict quantifications of the global vGAT staining intensity (**e**) and the vGAT-positive puncta density (**f**) as two measures of synapse density. Data are means ± SEM; numbers of sections/mice are indicated in the bars. Statistical significance was assessed using two-sided Student's t-test and Two-way ANOVA (non-significant comparisons are not indicated). Scale bars: 1 mm (**a**), 0.2 mm (**b**) and 10 µm (**c**).

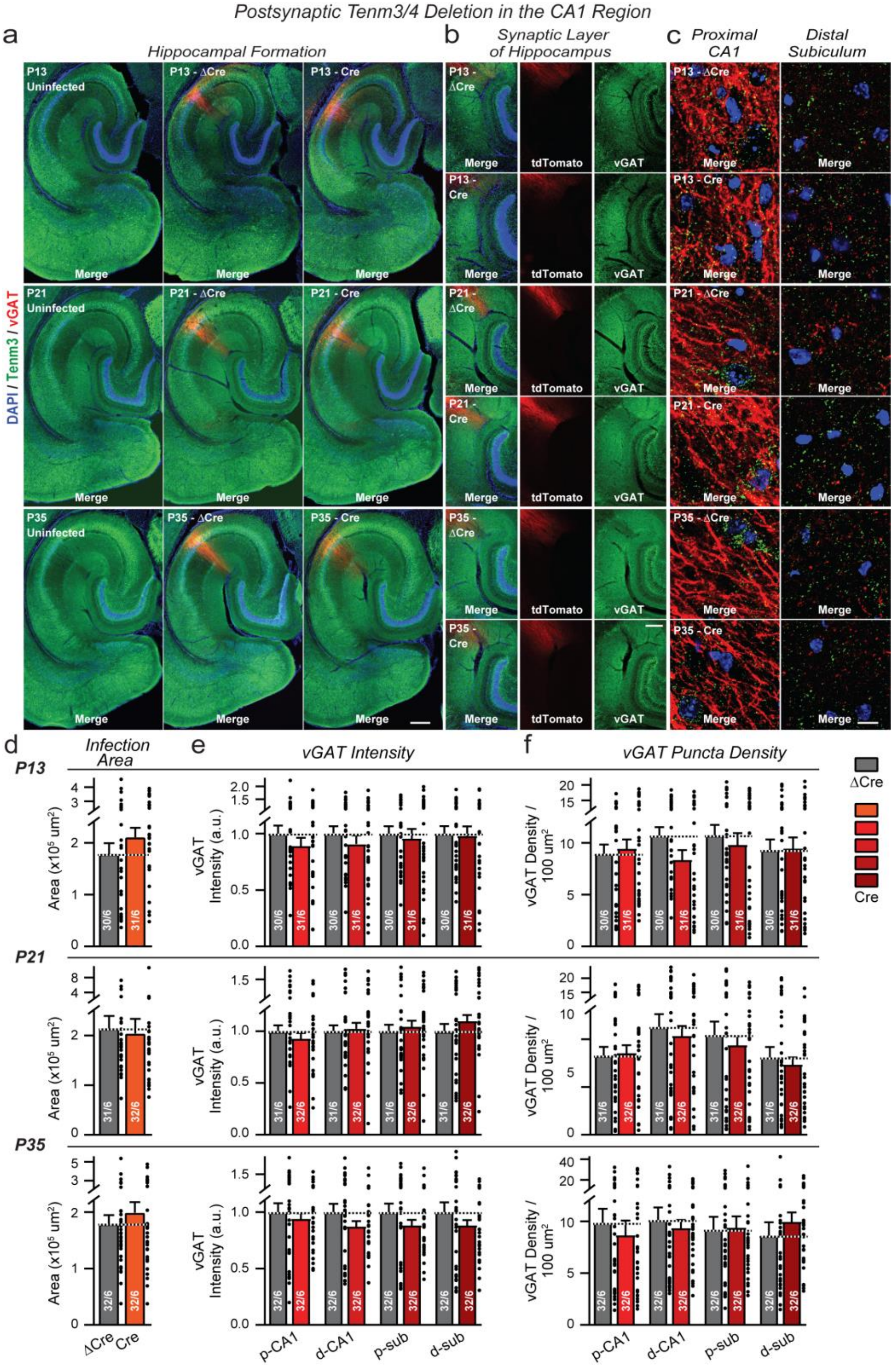

Supplementary figure 8: Inactivation of Tenm3 and Tenm4 expression in CA1 neurons has no effect on inhibitory synapse density in the CA1 and subiculum (related to Fig. 4). All data were from mice whose CA1 neurons were infected at P0 with AAVs expressing ΔCre (control) or Cre. All sections were stained for vGAT as a presynaptic marker of inhibitory synapses. **a** Representative overview images of hippocampal sections from uninfected mice (left) and mice infected with AAVs expressing ΔCre (center) or Cre (right), examined at P13 (top row), P21 (middle row), or P35 (bottom row). **b** Representative low-resolution images of vGAT-stained hippocampal sections corresponding to the overviews

shown in **a**. **c** Representative high-resolution images of vGAT staining in the S. moleculare-lacunosum of the proximal CA1 (left) and the molecular layer of distal subiculum (right) at P13, P21, and P35. **d** Quantifications showing that the CA1 region areas infected with ΔCre- and Cre-expressing AAVs, as measured via the tdTomato signal, were not significantly different at P13, P21, and P35. **e, f** Postsynaptic deletion of Tenm3/4 in CA1 region neurons causes no change in the overall density of inhibitory vGAT-positive synapses in the indicated areas. Summary graphs depict quantifications of the global vGAT staining intensity (**e**) and the vGAT-positive puncta density (**f**) as two measures of synapse density. Data are means ± SEM; numbers of sections/mice are indicated in the bars. Statistical significance was assessed using two-sided Student's t-test and Two-way ANOVA (non-significant comparisons are not indicated). Scale bars: 1 mm (**a**), 0.2 mm (**b**) and 10 µm (**c**).

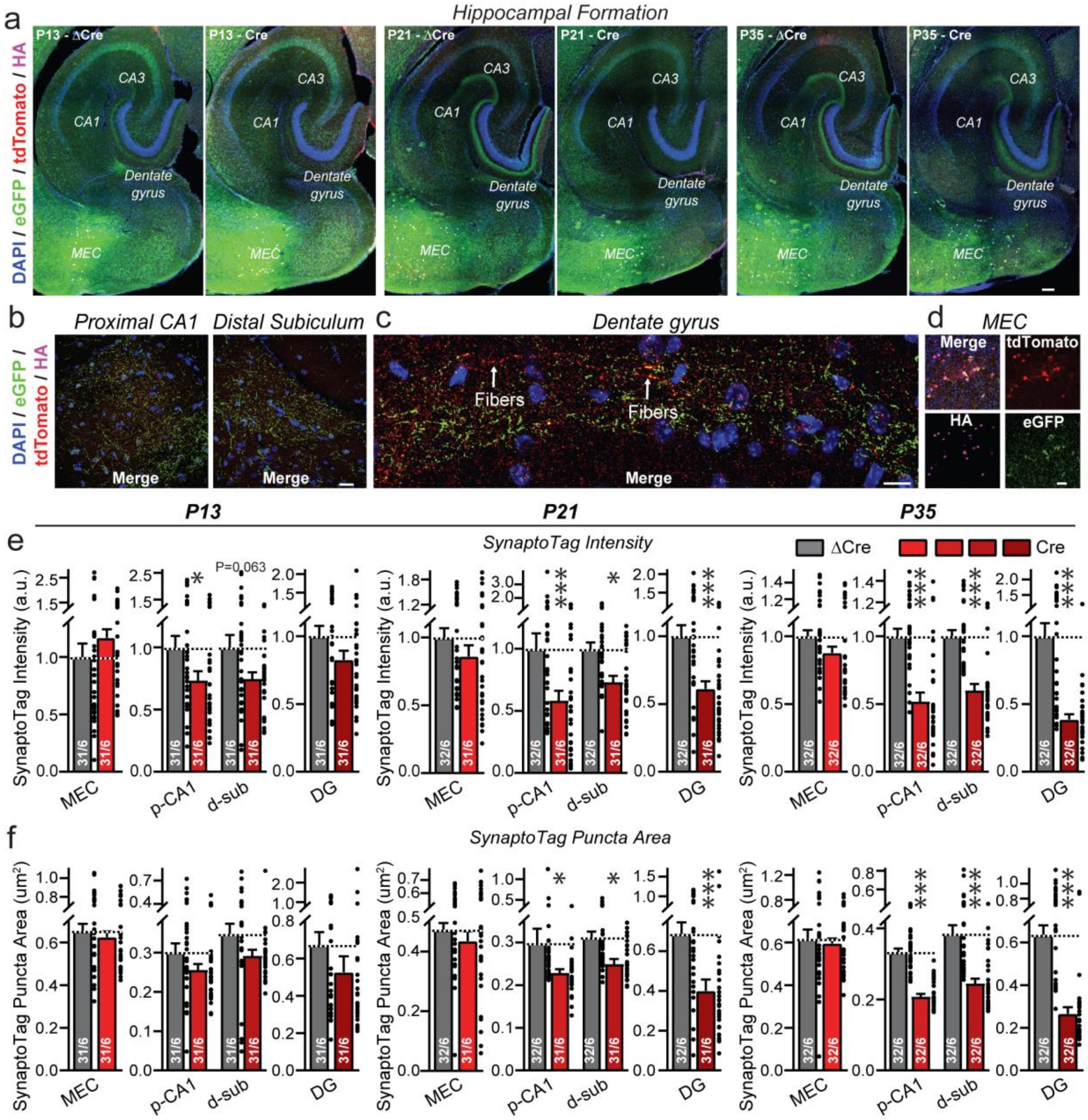

Supplementary figure 9: Further data using SynaptoTag labeling demonstrating that presynaptic inactivation of Tenm3 and Tenm4 expression in the medial entorhinal cortex neurons severely suppresses formation of output synapses formed by MEC neurons in the CA1 region, subiculum, and dentate gyrus (related to Fig. 6). a Representative images of hippocampal sections after sparse infection in MEC neurons of Tenm3/4 DcKO mice with ΔCre (left in each group) or Cre (right in each group) and SynaptoTag expression at P0 and analysis at P13 (left two), P21 (middle two), or P35 (right two). **b-d** Representative high-resolution images show synaptic targets for medial entorhinal cortex (MEC) neurons using SynaptoTag tracing approach. **b**, left: proximal CA1, right, distal subiculum; **c**, dentate gyrus (DG); **d**, medial entorhinal cortex (MEC). Magenta HA marks Cre or ΔCre, Red tdTomate labeling marks axonal fibers, whereas green eGFP labeling marks synapses projecting from the MEC, and a blue labeling nuclear DAPI. **e** SynaptoTag intensity of MEC, p-CA1, d-sub, and DG were analyzed between control mice (ΔCre) and test (Cre) mice at P13 (left), P21 (middle), or P35 (right). Presynaptic deletion of Tenm3/4 in MEC neurons decreases the SynaptoTag density in p-CA1, d-sub, and dentate gyrus (DG), but not MEC itself. SynaptoTag intensity of test mice (Cre) was normalized to control mice (ΔCre). **f** SynaptoTag puncta area of MEC, p-CA1, d-sub, and DG were analyzed between

control mice (ΔCre) and test (Cre) mice at P13 (left), P21 (middle), or P35 (right). Presynaptic deletion of Tenm3/4 in MEC neurons decreases the SynaptoTag puncta area in p-CA1, d-sub, and dentate gyrus (DG), but not MEC. Bar graphs show means ± SEM; numbers of sections/mice are indicated in the bars. Statistical significance was assessed using two-sided Student's t-test and Two-way ANOVA (*, $p<0.05$; **, $p<0.01$; ***, $p<0.001$). Scale bars: 0.5 mm (**a**), 20 µm (**b**), 10 µm (**c**) and 0.1 mm (**d**).

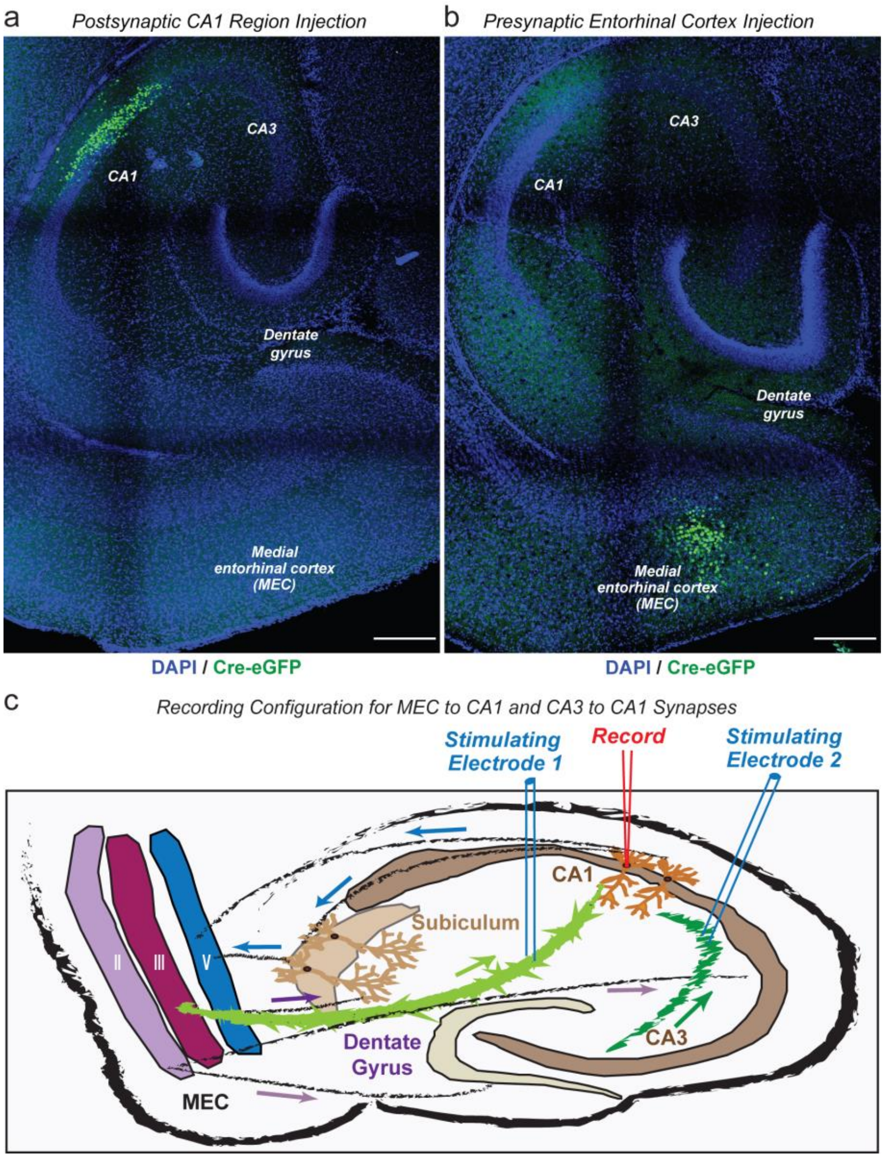

Supplementary figure 10: Further data for selective deletions of both Tenm3 and Tenm4 from the MEC or CA1 in Tenm3/4 double cKO mice to achieve exclusively pre- or postsynaptic ablation of Tenm3 and Tenm4 expression for the electrophysiological recording (related to Fig. 8 and 10). a, b, Representative images of a horizontal section of the hippocampal formation of a Tenm3/4 DcKO mouse at P18 whose CA1 neurons in **a** or MEC neurons in **b** was infected with Cre- and nls-eGFP-expressing AAVs at P0. **c,** Diagram of the electrophysiological recording configuration in acute slices with AAVs injections. Scale bars: 1 mm.
