# Supplementary material for: Teneurins assemble into presynaptic nanoclusters that promote synapse formation via postsynaptic non-teneurin ligands
Source: Nat Commun. 2022 Apr 28;13:2297. doi: 10.1038/s41467-022-29751-1 (PMC9050732; doi:10.1038/s41467-022-29751-1)
Supplement: Supplementary file 2 — Reporting Summary [file 41467_2022_29751_MOESM2_ESM.pdf]

nature portfolio

Corresponding author(s): Xuchen Zhang and Thomas C. Südhof

Last updated by author(s): Feb 28, 2022

Reporting Summary

Nature Portfolio wishes to improve the reproducibility of the work that we publish. This form provides structure for consistency and transparency in reporting. For further information on Nature Portfolio policies, see our Editorial Policies and the Editorial Policy Checklist.

Statistics

For all statistical analyses, confirm that the following items are present in the figure legend, table legend, main text, or Methods section.

n/a	Confirmed	
☐	☒	The exact sample size (n) for each experimental group/condition, given as a discrete number and unit of measurement
☐	☒	A statement on whether measurements were taken from distinct samples or whether the same sample was measured repeatedly
☐	☒	The statistical test(s) used AND whether they are one- or two-sided<br>*Only common tests should be described solely by name; describe more complex techniques in the Methods section.*
☐	☒	A description of all covariates tested
☐	☒	A description of any assumptions or corrections, such as tests of normality and adjustment for multiple comparisons
☐	☒	A full description of the statistical parameters including central tendency (e.g. means) or other basic estimates (e.g. regression coefficient) AND variation (e.g. standard deviation) or associated estimates of uncertainty (e.g. confidence intervals)
☐	☒	For null hypothesis testing, the test statistic (e.g. F, t, r) with confidence intervals, effect sizes, degrees of freedom and P value noted<br>*Give P values as exact values whenever suitable.*
☒	☐	For Bayesian analysis, information on the choice of priors and Markov chain Monte Carlo settings
☒	☐	For hierarchical and complex designs, identification of the appropriate level for tests and full reporting of outcomes
☐	☒	Estimates of effect sizes (e.g. Cohen's d, Pearson's r), indicating how they were calculated

Our web collection on statistics for biologists contains articles on many of the points above.

Software and code

Policy information about availability of computer code

Data collection	Nikon confocal microscope (AR 5.30.04) (A1RSi)<br>Vutara SR 352 (6.04.19) (Bruker Nanosurfaces, Inc., Madison, WI)<br>the ChemiDoc Gel Imaging System (6.0.1) (Bio-Rad)
Data analysis	Nikon NIS-Elements (5.30.04) Nikon RRID:SCR_014329<br>ImageJ (bundled with 64-bit Java 1.8.0_172), NIH, RRID: SCR_003070<br>GraphPad Prism 9, GraphPad Prism, RRID:SCR_002798R2019a<br>Matlab R2019a, The MathWorks, RRID:SCR_001622

For manuscripts utilizing custom algorithms or software that are central to the research but not yet described in published literature, software must be made available to editors and reviewers. We strongly encourage code deposition in a community repository (e.g. GitHub). See the Nature Portfolio guidelines for submitting code & software for further information.

Data

Policy information about availability of data

All manuscripts must include a data availability statement. This statement should provide the following information, where applicable:

- Accession codes, unique identifiers, or web links for publicly available datasets
- A description of any restrictions on data availability
- For clinical datasets or third party data, please ensure that the statement adheres to our policy

All relevant data and code supporting the findings of this study are available from the corresponding authors upon request. Source data are provided with this

paper. Heat maps in Extended Data Fig. 3 of NAUC (normalized area under the curve) expression values were obtained from the ASCOT (Alternative Splicing & Gene Expression Summaries of Public RNA-Seq Data) database (http://ascot.cs.jhu.edu/).

Field-specific reporting

Please select the one below that is the best fit for your research. If you are not sure, read the appropriate sections before making your selection.

☒ Life sciences ☐ Behavioural & social sciences ☐ Ecological, evolutionary & environmental sciences

For a reference copy of the document with all sections, see nature.com/documents/nr-reporting-summary-flat.pdf

Life sciences study design

All studies must disclose on these points even when the disclosure is negative.

Sample size	We did not perform power analysis to predetermine sample sizes, but our sample sizes are similar to those generally employed in the field. (ref. 6, 7, 33, 35, 56, 57, 62).
Data exclusions	For all experiments, the animals' brain were processed for histology to confirm virus infection sites. Data will be excluded if the infection sites were off from the desired nuclei. The exclusion criteria were pre-determined before any experiment.
Replication	The number of independently replicated experiments is described in the figure legends and text. All attempts to replicate the results were successful.
Randomization	For P0 pups injection, littermates were randomly coded and allocated into experimental and control groups, received the same injections of ΔCre or Cre were encoded in AAV or Lentiviruses. All the samples were also randomized collected into two groups and analyzed blindly, as explained in the methods.<br>For all other experiments, samples were also collected, coded, and analyzed blindly, as indicated in the data analyses part.
Blinding	Both the data collection and analysis process was blinded. Each mouse was coded with a unique ID and the type of virus it received was blindly injected by the experimenters.

Behavioural & social sciences study design

All studies must disclose on these points even when the disclosure is negative.

Study description	*Briefly describe the study type including whether data are quantitative, qualitative, or mixed-methods (e.g. qualitative cross-sectional, quantitative experimental, mixed-methods case study).*
Research sample	*State the research sample (e.g. Harvard university undergraduates, villagers in rural India) and provide relevant demographic information (e.g. age, sex) and indicate whether the sample is representative. Provide a rationale for the study sample chosen. For studies involving existing datasets, please describe the dataset and source.*
Sampling strategy	*Describe the sampling procedure (e.g. random, snowball, stratified, convenience). Describe the statistical methods that were used to predetermine sample size OR if no sample-size calculation was performed, describe how sample sizes were chosen and provide a rationale for why these sample sizes are sufficient. For qualitative data, please indicate whether data saturation was considered, and what criteria were used to decide that no further sampling was needed.*
Data collection	*Provide details about the data collection procedure, including the instruments or devices used to record the data (e.g. pen and paper, computer, eye tracker, video or audio equipment) whether anyone was present besides the participant(s) and the researcher, and whether the researcher was blind to experimental condition and/or the study hypothesis during data collection.*
Timing	*Indicate the start and stop dates of data collection. If there is a gap between collection periods, state the dates for each sample cohort.*
Data exclusions	*If no data were excluded from the analyses, state so OR if data were excluded, provide the exact number of exclusions and the rationale behind them, indicating whether exclusion criteria were pre-established.*
Non-participation	*State how many participants dropped out/declined participation and the reason(s) given OR provide response rate OR state that no participants dropped out/declined participation.*
Randomization	*If participants were not allocated into experimental groups, state so OR describe how participants were allocated to groups, and if allocation was not random, describe how covariates were controlled.*

Ecological, evolutionary & environmental sciences study design

All studies must disclose on these points even when the disclosure is negative.

Study description	*Briefly describe the study. For quantitative data include treatment factors and interactions, design structure (e.g. factorial, nested, hierarchical), nature and number of experimental units and replicates.*
Research sample	*Describe the research sample (e.g. a group of tagged Passer domesticus, all Stenocereus thurberi within Organ Pipe Cactus National Monument), and provide a rationale for the sample choice. When relevant, describe the organism taxa, source, sex, age range and any manipulations. State what population the sample is meant to represent when applicable. For studies involving existing datasets, describe the data and its source.*
Sampling strategy	*Note the sampling procedure. Describe the statistical methods that were used to predetermine sample size OR if no sample-size calculation was performed, describe how sample sizes were chosen and provide a rationale for why these sample sizes are sufficient.*
Data collection	*Describe the data collection procedure, including who recorded the data and how.*
Timing and spatial scale	*Indicate the start and stop dates of data collection, noting the frequency and periodicity of sampling and providing a rationale for these choices. If there is a gap between collection periods, state the dates for each sample cohort. Specify the spatial scale from which the data are taken*
Data exclusions	*If no data were excluded from the analyses, state so OR if data were excluded, describe the exclusions and the rationale behind them, indicating whether exclusion criteria were pre-established.*
Reproducibility	*Describe the measures taken to verify the reproducibility of experimental findings. For each experiment, note whether any attempts to repeat the experiment failed OR state that all attempts to repeat the experiment were successful.*
Randomization	*Describe how samples/organisms/participants were allocated into groups. If allocation was not random, describe how covariates were controlled. If this is not relevant to your study, explain why.*
Blinding	*Describe the extent of blinding used during data acquisition and analysis. If blinding was not possible, describe why OR explain why blinding was not relevant to your study.*

Did the study involve field work? ☐ Yes ☐ No

Field work, collection and transport

Field conditions	*Describe the study conditions for field work, providing relevant parameters (e.g. temperature, rainfall).*
Location	*State the location of the sampling or experiment, providing relevant parameters (e.g. latitude and longitude, elevation, water depth).*
Access & import/export	*Describe the efforts you have made to access habitats and to collect and import/export your samples in a responsible manner and in compliance with local, national and international laws, noting any permits that were obtained (give the name of the issuing authority, the date of issue, and any identifying information).*
Disturbance	*Describe any disturbance caused by the study and how it was minimized.*

Reporting for specific materials, systems and methods

We require information from authors about some types of materials, experimental systems and methods used in many studies. Here, indicate whether each material, system or method listed is relevant to your study. If you are not sure if a list item applies to your research, read the appropriate section before selecting a response.

Materials & experimental systems

n/a	Involved in the study	
☐	☒	Antibodies
☐	☒	Eukaryotic cell lines
☒	☐	Palaeontology and archaeology
☐	☒	Animals and other organisms
☒	☐	Human research participants
☒	☐	Clinical data
☒	☐	Dual use research of concern

Methods

n/a	Involved in the study	
☒	☐	ChIP-seq
☒	☐	Flow cytometry
☒	☐	MRI-based neuroimaging

Antibodies

Antibodies used	Primary antibodies: Guinea pig anti-vGluT1, Millipore Cat# AB5905, Rabbit anti-vGluT1, TCS Cat# YZ6089, Guinea pig anti-vGAT, Millipore Cat# AB5062P, Guinea pig anti-vGAT, Synaptic Systems Cat# 131004, Mouse anti-Bassoon, Abcam Cat# 82958, Mouse anti-Homer1, Synaptic Systems Cat# 160011, Rabbit anti-Homer1, Synaptic Systems Cat# 160003, Chicken anti-GFP, Aves Labs Cat# 1020, Chicken anti-GFP, Invitrogen Cat# A10262, Rat anti-mCherry, Invitrogen Cat# M11217, Rabbit anti-HA, Cell Signaling Technologies Cat# 3724, Rabbit anti-Teneurin3 (IC, a gift from Liqun Luo).<br><br>Secondary antibodies: Goat anti-Chicken IgG (H+L) Secondary Antibody, Alexa Fluor 488, Invitrogen Cat# A11039, Goat anti-Guinea Pig IgG (H+L) Highly Cross-Adsorbed Secondary Antibody, Alexa Fluor 647, Invitrogen Cat# A-21450, Goat anti-Rat IgG (H+L) Cross-Adsorbed Secondary Antibody, Alexa Fluor 546, Invitrogen Cat# A-11081, Goat anti-Rabbit IgG (H+L) Highly Cross-Adsorbed Secondary Antibody, Alexa Fluor 647, Invitrogen Cat# A-21245, Highly cross-adsorbed donkey anti-mouse IgG (H+L) secondary antibody CF568, Biotium, 20105.
Validation	All antibodies have been validated in previous papers (ref. 29, 35, 47, 57, 62).

Eukaryotic cell lines

Policy information about cell lines

Cell line source(s)	HEK 293T (CRL11268)
Authentication	HEK 293T cells were directly purchased from ATCC. ATCC determines the biosafety level of a material based on our risk assessment as guided by the current edition of Biosafety in Microbiological and Biomedical Laboratories (BMBL), U.S. Department of Health and Human Services.
Mycoplasma contamination	Cell lines were tested negative for mycoplasma contamination using the fluorochrome Hoechst DNA stain and the direct culture method.
Commonly misidentified lines<br>(See ICLAC register)	None of the cell lines used is listed as commonly misidentified.

Animals and other organisms

Policy information about studies involving animals; ARRIVE guidelines recommended for reporting animal research

Laboratory animals	Tenm3 cKO mice were described previously (ref 29, JAX stock # 031705), and Tenm4 cKO mice were a gift from Liqun Luo. Tenm3 and Tenm4 cKO mice were crossed to generate Tenm3/4 DcKO mice. Mice were weaned at 20-21 days of age and group-housed (less than 5 mice per cage) on a 12 h light/dark cycle with food and water ad libidum at room temperature and 40–60% humidity, both male and female animals were used for all experiments. All studies used littermate male or female mice with the same age. P0 pups were used for virus injections and were analyzed at P13, P21 and P35 after virus injection. The same injected mice at P21 and P35 were re-infected rabies complementing AAVs for Monosynaptic retrograde rabies viral tracing experiments (Figs 7 and 9)
Wild animals	No wild animals were involved in this study.
Field-collected samples	This study did not involve field-collected samples.
Ethics oversight	All animal procedures conformed to the National Institutes of Health Guidelines for the Care and Use of Laboratory Animals and were approved by Administrative Panel on Laboratory Animal Care at Stanford University.

Note that full information on the approval of the study protocol must also be provided in the manuscript.

ChIP-seq

Data deposition

☐ Confirm that both raw and final processed data have been deposited in a public database such as GEO.

☐ Confirm that you have deposited or provided access to graph files (e.g. BED files) for the called peaks.

Data access links<br>*May remain private before publication.*	*For "Initial submission" or "Revised version" documents, provide reviewer access links. For your "Final submission" document, provide a link to the deposited data.*
Files in database submission	*Provide a list of all files available in the database submission.*
Genome browser session<br>(e.g. UCSC)	*Provide a link to an anonymized genome browser session for "Initial submission" and "Revised version" documents only, to enable peer review. Write "no longer applicable" for "Final submission" documents.*

Methodology

Replicates	*Describe the experimental replicates, specifying number, type and replicate agreement.*

Sequencing depth	*Describe the sequencing depth for each experiment, providing the total number of reads, uniquely mapped reads, length of reads and whether they were paired- or single-end.*
Antibodies	*Describe the antibodies used for the ChIP-seq experiments; as applicable, provide supplier name, catalog number, clone name, and lot number.*
Peak calling parameters	*Specify the command line program and parameters used for read mapping and peak calling, including the ChIP, control and index files used.*
Data quality	*Describe the methods used to ensure data quality in full detail, including how many peaks are at FDR 5% and above 5-fold enrichment.*
Software	*Describe the software used to collect and analyze the ChIP-seq data. For custom code that has been deposited into a community repository, provide accession details.*
